# Nitrate transiently rewires transcription in developing xylem of *Populus* wood to promote cell expansion via CRF4

**DOI:** 10.1093/plcell/koag281

**Published:** 2026-09-18

**Authors:** Shruti Choudhary, Anna Renström, Theerarat Kochakarn, Pal Miskolczi, Edoardo Piombo, Maxime Chantreau, Jeny Jose, Sandra Jämtgård, András Gorzsás, Johan Henriksson, Hannele Tuominen

**Affiliations:** Umeå Plant Science Centre, Department of Forest Genetics and Plant Physiology, Swedish University of Agricultural Sciences, 90183 Umeå, Sweden; Umeå Plant Science Centre, Department of Forest Genetics and Plant Physiology, Swedish University of Agricultural Sciences, 90183 Umeå, Sweden; Umeå Plant Science Centre, Department of Plant Physiology, Umeå University, 90187 Umeå, Sweden; Umeå Plant Science Centre, Department of Forest Genetics and Plant Physiology, Swedish University of Agricultural Sciences, 90183 Umeå, Sweden; Umeå Plant Science Centre, Department of Plant Physiology, Umeå University, 90187 Umeå, Sweden; Umeå Plant Science Centre, Department of Forest Genetics and Plant Physiology, Swedish University of Agricultural Sciences, 90183 Umeå, Sweden; Umeå Plant Science Centre, Department of Forest Genetics and Plant Physiology, Swedish University of Agricultural Sciences, 90183 Umeå, Sweden; Department of Forest Ecology and Management, Swedish University of Agricultural Sciences, 90183 Umeå, Sweden; Science for Life Laboratory, Swedish University of Agricultural Sciences, 90183 Umeå, Sweden; Department of Chemistry, Umeå University, 90187 Umeå, Sweden; Department of Molecular Biology, Umeå University, 90187 Umeå, Sweden; Integrated Science Lab (Icelab), Umeå University, 90187 Umeå, Sweden; Science for Life Laboratory (SciLifeLab), Umeå University, 90187 Umeå, Sweden; Umeå Centre for Microbial Research (UCMR), Umeå University, 90187 Umeå, Sweden; Umeå Plant Science Centre, Department of Forest Genetics and Plant Physiology, Swedish University of Agricultural Sciences, 90183 Umeå, Sweden

## Abstract

Nitrate functions both as a nutrient and a signaling molecule, but how nitrate is perceived and translated into developmental outputs within woody tissues is unknown. Here, we show that developing xylem in hybrid aspen (*Populus tremula × P. tremuloides*) mounts a rapid, cell-type-specific transcriptional response to nitrate that promotes xylem cell expansion. To isolate local nitrate signaling in wood-forming tissues, we delivered nitrate directly into the xylem sap and profiled transcriptomic responses from 2 to 48 h after treatment. Bulk RNA sequencing revealed a transient response peaking within 2 to 4 h. Single-cell RNA sequencing at the peak response resolved nitrate signaling across developing xylem cell types and showed that nitrate induced cell-type-specific programs linked to nitrogen transport, cell wall remodeling, and developmental progression, while repressing lignin-biosynthetic genes. These data indicate that nitrate acts transiently in wood-forming tissues to fine-tune early xylem development. Among nitrate-responsive transcription factors, CYTOKININ RESPONSE FACTOR 4 (CRF4) emerged as a candidate regulator of this response. *CRF4* overexpression in hybrid aspen stimulated xylem cell expansion, whereas simultaneous knockout of *CRF4* and its paralog, *CRF3*, significantly attenuated the nitrate-induced expansion. Together, our findings identify a local nitrate signaling module in developing wood and establish CRF4 as a component linking nitrate sensing to xylem cell expansion.

## Introduction

Nitrate is critical as a nutrient, being a major form of nitrogen utilized by plants (Crawford and Forde 2002). Nitrate availability often varies in the soil and can be a limiting factor to plant growth and development. To cope with fluctuations in nitrate availability, plants have evolved multiple mechanisms to coordinate nitrate uptake, transport, and distribution in ways that optimize nutrient use and energy expenditure. In poplar and other woody species, this coordination is particularly demanding because of the large size and architectural complexity of the organism. Nitrate is taken up by the roots and transported to the shoot of trees through the xylem, where it can be assimilated in both belowground and aboveground tissues depending on species, developmental stage, and nitrogen availability (Black et al. 2002; Castro-Rodríguez et al. 2017). Transcriptomic studies have suggested that nitrate assimilation is not confined to roots and leaves, but may also occur in stem tissues, including developing xylem and associated parenchyma cells (Zhang et al. 2025). However, the extent to which nitrate is actively retrieved from the xylem sap along its transport route, and the molecular components mediating this process in woody tissues, remain largely unknown.

Nitrate is distinct from other nitrogen sources as it also acts as a signaling molecule and can rapidly induce gene expression changes after its perception. The machinery of nitrate acquisition, sensing, and signaling is well documented, including NRT1.1/NPF6.3 as the nitrate transceptor, NIN-like protein (NLP) gene family members as sensors, and several other transcriptional regulators mediating the response (Ho et al. 2009; Rubin et al. 2009; Varala et al. 2018; Fredes et al. 2019; Liu et al. 2022). Nitrate-elicited molecular events are time-dependent as the expression change for different sets of genes can be observed within a few minutes to several days after its application (Scheible et al. 2004; Krouk et al. 2010; Krapp et al. 2011; Alvarez et al. 2012). Nitrate rapidly triggers genes responsible for its own transport and assimilation, and influences several other genes considered vital in signaling and primary nitrate response in *Arabidopsis* (Krouk et al. 2010; Bouguyon et al. 2015; Liu et al. 2017; Varala et al. 2018; Alvarez et al. 2020).

Another enticing fact is that nitrate uptake and response is not just systemic communication across different organs but also comprises of a local signal that acts at the cellular level (Delgado et al. 2024). Different cell types with their specialized functions can respond to nitrate signal in a unique manner, allowing plants to tweak their responses in a cell-specific manner. Nitrate has been shown to induce coordinated yet distinct cellular responses in different cell types of *Arabidopsis* roots. Cortex and pericycle cells subjected to 48-h ammonium nitrate treatment showed specific changes in gene expression that were, however, governed by a common set of transcription factors (Walker et al. 2017). More recently, nitrate-responsive transcriptomic changes were reported as early as 12 min in the outermost cells of the root, followed by spreading of the response to all other cell types of roots (Contreras-López et al. 2022). These changes align well with real-time in vivo monitoring of nitrate levels using a genetically encoded biosensor, which demonstrated the entrance of exogenous nitrate into most of the *Arabidopsis* root cells within 5 min (Chen et al. 2022). Together, these studies reveal spatiotemporal specificity in nitrate sensing, uptake, and assimilation, likely serving efficient adaptation toward prevailing nitrate conditions.

Much of the research on nitrate signaling has focused on roots, while less is known about cellular responses to nitrate in the shoot. Trees provide a particularly compelling system in this regard because their shoots contain the majority of terrestrial plant biomass and are the primary site of wood formation. In *Populus*, it has been reported that fertilization with nitrate stimulates the activity of the apical and the vascular meristems, and influences differentiation of xylem elements in the wood (Poovaiah et al. 2019; Renström et al. 2024). In particular, nitrate stimulates the expansion of xylem elements in developing wood (Renström et al. 2024; Xu et al. 2024). However, these studies have captured only a snapshot of a long-term exposure of nitrate in bulk samples and do therefore not give information on temporal or spatial regulation of xylem cell expansion. In this study, we aimed to provide molecular-level insights into the early events of xylem development after nitrate application by dissecting the transcriptional response with an unprecedented spatial and temporal resolution in woody tissues of hybrid aspen (*P. tremula* × *P. tremuloides*) trees. To narrow down the observed nitrate responses exclusively in wood, we designed a strategy to bypass the root-to-shoot communication of the nitrate signal by applying nitrate directly into the xylem sap. We performed a time-resolved transcriptomic analysis from 2 to 48 h after the treatment to capture the progression of nitrate signaling in the developing xylem. Genes associated with xylem cell expansion were induced 2 h after nitrate application, which led us to profile the xylem transcriptome on a cellular level at this time point. scRNA-seq analysis revealed strongest response to nitrate in the xylem rays as well as fibers and vessels at their earliest developmental stage. By integrating temporal and cellular transcriptomic data, we identified transcriptional networks specific to distinct xylem cell types and uncovered candidate regulators of nitrate-mediated wood development. Among these, we identify CYTOKININ RESPONSE FACTOR 4 (CRF4) as a key regulator linking nitrate signaling to xylem cell expansion.

## Results

### The timing of nitrate response is dynamic in woody tissues

Nitrate elicits fast metabolic and developmental changes in plants. To determine the timing of nitrate response exclusively in woody tissues, we injected 5 mM potassium nitrate (KNO_3_) or potassium chloride (KCl, control) directly into the xylem sap of nitrogen-starved hybrid aspen (*Populus tremula* × *P. tremuloides*) trees (Fig. 1). The total amount of applied nitrate corresponded to 0.14 mg of nitrogen, which is within the physiological range of nitrate found in xylem sap (Hu and Guy 2020). The injected solutions were taken up within 2 h. Transcriptomic and morphological changes in the developing xylem tissues were followed over a time course from 2 to 48 h after the injections (Fig. 1, a and b). Parallel experiments with injection of acid fuchsin stain demonstrated that the whole woody tissue was likely to receive the injected solution 60 cm from the injection point within 2 h (Fig. 1c). Samples for transcriptional analysis were therefore collected 40 to 60 cm from the injection point (Fig. 1c). Finally, we examined the effect of nitrate application on the size of developing xylem cells. Cell size was determined by isolating protoplasts from the surface of the developing secondary xylem following bark removal and enzymatic cell wall digestion (Fig. 1, d to f). This approach enabled measurement of the entire cell volume specifically in the most recently developed xylem cells. Nitrate treatment significantly increased the average size of developing xylem elements after 48 h compared with the control (Fig. 1g).

**Figure 1:**
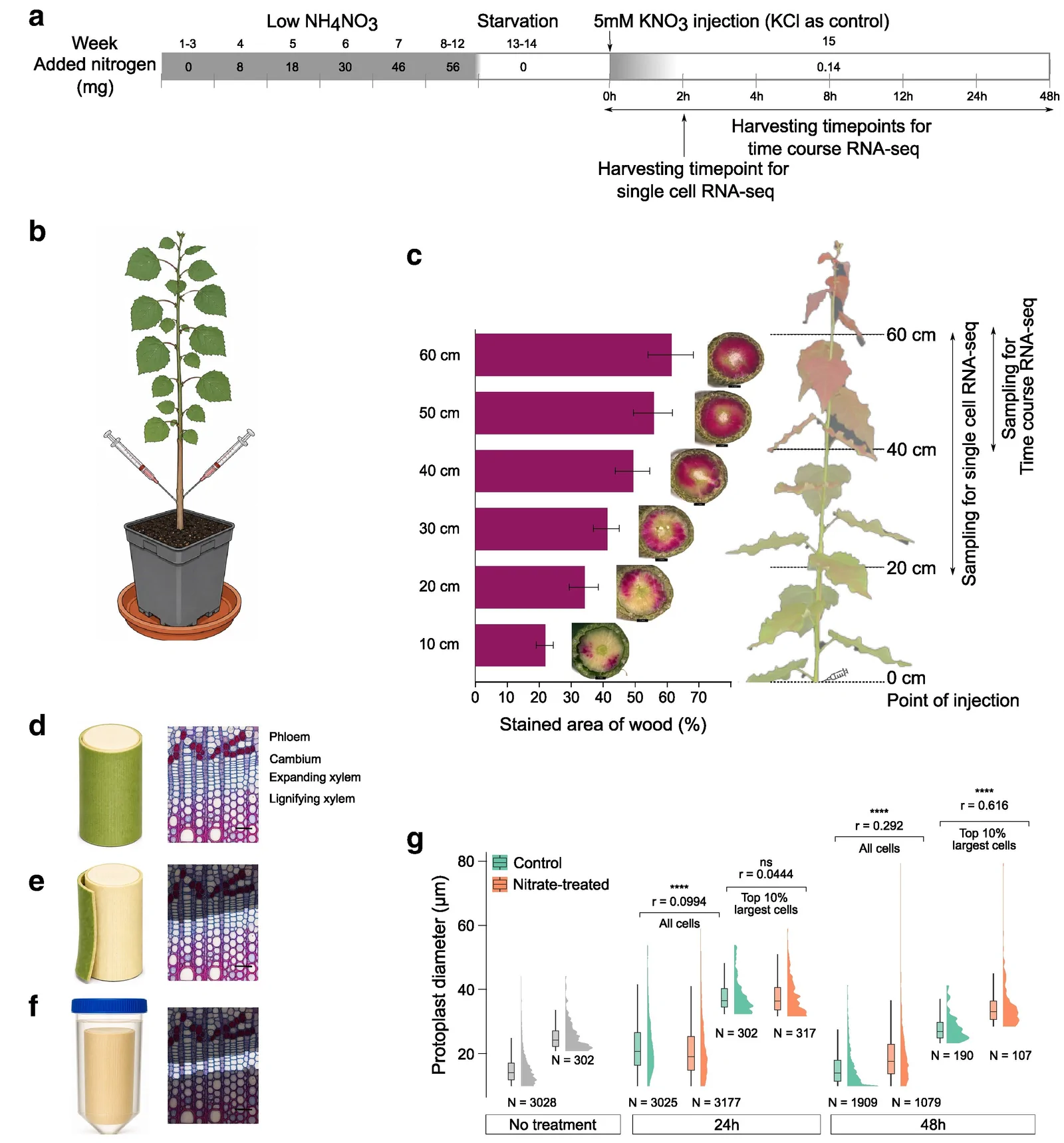
Experimental setup for the nitrate treatment in hybrid aspen trees. a) The timeline of nitrate treatment. After 14 wk of growth (first 12 wk under low-nitrogen [NH_4_NO_3_] conditions followed by 2 wk of nitrogen depletion), 2 mL of 5 mM KNO_3_ solution (corresponding to ∼0.14 mg of total nitrogen) was injected directly into the xylem sap of the stem. Injection of 2 mL 5 mM KCl solution was used as a control. Stem samples were harvested at 0, 2, 4, 8, 12, 24, and 48 h after injections for the time course and at 2 h after injections for single-cell transcriptome profiling. b) Injection into the xylem sap. Two syringes (à 1 mL) were used to inject the KNO_3_ or KCl solutions into the xylem sap at the base of the stem. c) Scheme of sampling for the RNA-seq. Stem samples were taken 40 to 60 cm and 20 to 60 cm above the point of injections for the time course and single-cell RNA-seq experiment, respectively. d to f) The procedure to isolate protoplasts from developing wood. d) Intact stem piece and a corresponding schematic view of the anatomy in transverse orientation. e) The bark is peeled resulting in a fracture between the diving cambial cells and the expanding xylem. A few cambial cells often remain on the surface of the wood. The tissues excluded by peeling are darkened in the schematic transverse section. f) The peeled stem pieces are incubated in a solution of hydrolytic enzymes to release the protoplasts from the surface of the exposed woody tissue. The 3 to 4 cell layers, typically releasing the protoplasts according to this procedure, are shown in light color. The schematic pictures are based on transverse sections from nitrogen-starved, nitrate-treated trees and modified with ChatGPT. The scale bar indicates 50 µm. g) The effect of nitrate treatment on the xylem cell size. The cell size (diameter) was analyzed in protoplasts isolated from developing stem tissues 24 and 48 h after treatment with KNO_3_ or KCl. Statistics were performed for all cells as well as the largest 10% of the protoplast population (shown alongside all cells), likely to represent the xylem vessels. *N* = number of protoplasts from a pool of 5 trees per treatment and time point. Differences in cell sizes were evaluated using a linear mixed-effects model (LMM) with estimated marginal means shown at 95% confidence intervals. Significance levels are ns (not significant) and *P*  *<* 0.001 (indicated with 3 asterisks). Because the large number of measured cells can yield highly significant *P*-values even for modest differences, treatment effects were additionally quantified using the rank-biserial correlation (*r*) calculated from Wilcoxon–Mann–Whitney comparisons. This nonparametric effect size measures the magnitude and direction of the shift in cell-size distributions independently of sample size and without assuming normality. Consistent results from both the LMM and the effect-size analysis provide stronger evidence that nitrate-induced changes in protoplast size are robust and biologically meaningful.

Principal component analysis (PCA) displayed clustering of transcriptome profiles mainly according to the different time points after nitrate application (Fig. 2a). The transcriptomes corresponding to time points between 2 and 12 h, for both the control (KCl) and nitrate (KNO_3_) treatments, differed from the 0 h time point, while the transcriptomes for the time points of 24 and 48 h were similar to the 0 h time point. The nitrate treatment influenced transcriptome profiles only in the early time points (Fig. 2a). Differential gene expression analysis revealed accordingly that 142, 372, 98, and 2 unique genes (differentially expressed genes [DEGs]) responded to nitrate after 2, 4, 8, and 12 h of nitrate treatment, respectively (Fig. 2b; Supplemental Data Set S1, a to d), while there was no significant change in gene expression afterwards. To determine whether the transient nitrate response resulted from the cessation of nitrate supply approximately 2 h after injection, when the supplied nitrate had been taken up by all trees, we analyzed gene expression in developing xylem tissues of trees exposed to a continuous supply of nitrate. After 72 h of continuous treatment with 5 mM KNO_3_ or KCl, only 4 DEGs were detected between treatments, indicating that continuous nitrate supply elicited virtually no transcriptional response in developing xylem tissues (Supplemental Data Set S2c). These results suggest that developing xylem cells respond to transient increases in nitrate availability but not to sustained supply of nitrate.

**Figure 2:**
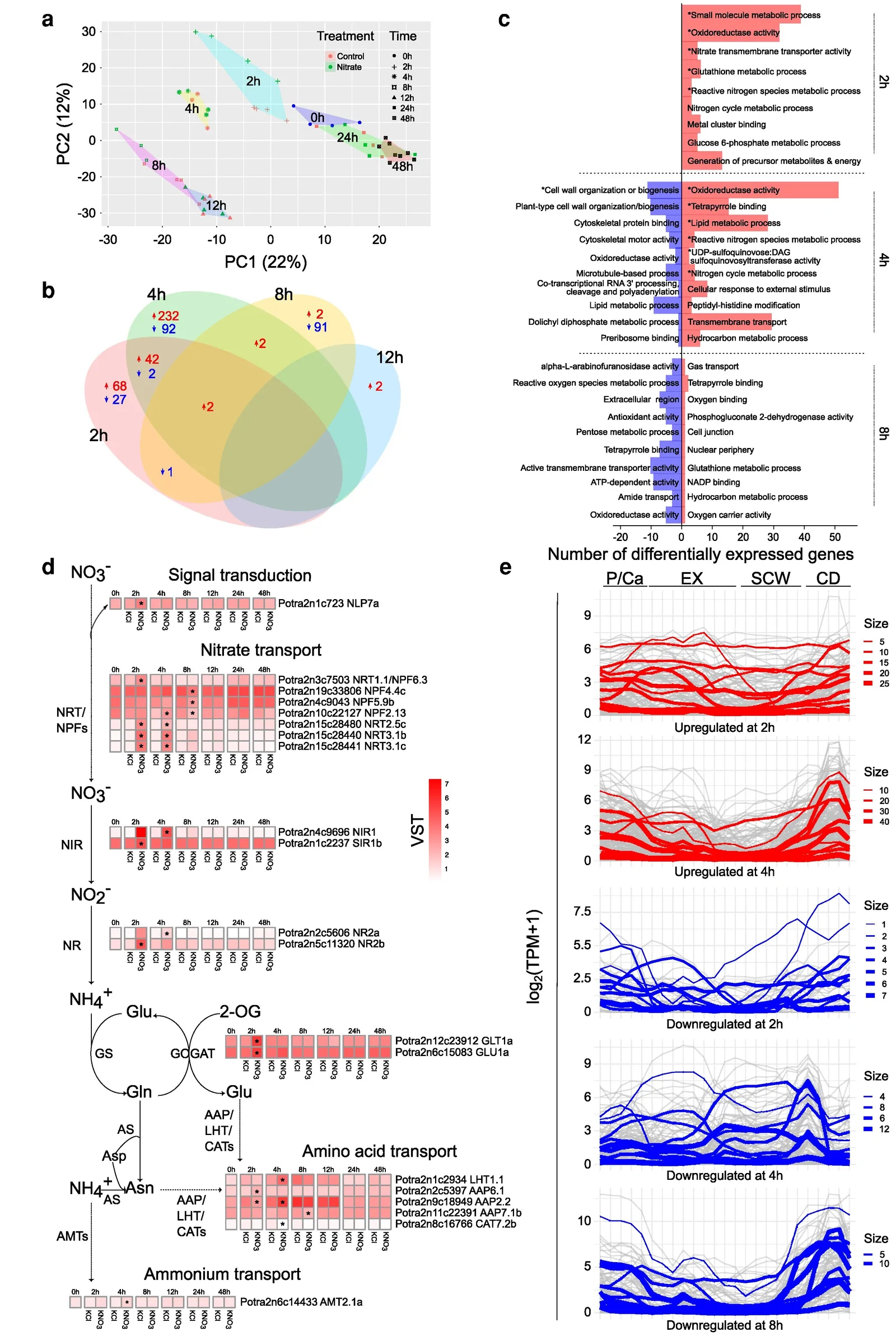
Time-course analysis of gene expression after nitrate treatment in developing xylem tissues of hybrid aspen stem. a) PCA of the RNA-seq data. Samples from the developing xylem tissues were collected at 0, 2, 4, 8, 12, 24, and 48 h after injection with either nitrate (KNO_3_) or control (KCl) solution. b) DEGs after nitrate treatment. The number of DEGs with absolute log_2_ fold change (LFC) > 1 and FDR < 0.05 are shown in pairwise comparisons between the control and the nitrate-treated samples at a specific time point after nitrate injection. See Supplemental Data Set S1, a to d, and Figure S1 for details. c) Top 10 most significant GO terms for the DEGs. Asterisks indicate significant terms. Gene annotations are based on the closest *Arabidopsis* homolog in TAIR. d) Expression of nitrogen transport, metabolism, and assimilation genes in response to nitrate treatment. The heatmap is based on VST values from RNA-seq of xylem tissues 0, 2, 4, 8, 12, 24, and 48 h after injection of nitrate (KNO_3_) or control (KCl) solutions. An asterisk indicates a significant change in gene expression (absolute LFC > 1 and FDR < 0.05) following nitrate treatment relative to control at the indicated time point. *P. tremula* gene annotations are based on Zhang et al. (2025). e) Cluster analysis of time-specific genes. DEGs were grouped into “time bins” based on their first appearance in the time series after nitrate treatment (Supplemental Data Set S1e). Hierarchical clustering was performed using log_2_(TPM+1)-transformed expression values obtained from the AspWood database. Up- and downregulation is indicated by red and blue, respectively, in panels (b), (c), and (e). P/Ca, phloem/cambium; Ex, expanding xylem; SCW, secondary cell wall formation; CD, cell death.

The gene ontology (GO) analysis of the DEGs in the time series revealed significant enrichment of terms related to *nitrate transmembrane transporter activity* and *nitrogen cycle metabolic process* (Fig. 2c). Accordingly, several nitrogen uptake and metabolism-related genes, including the homologs of the *NRT1.1/NPF6.3* and *NLP7*, were differentially regulated by nitrate (Fig. 2d).

All 29,591 genes expressed in the time-course experiment were used to create a whole-transcriptome co-expression network with 10 clusters and 28,821 nodes (genes) (Supplemental Data Set S1, f and g). A subnetwork was created with DEGs that were transcription factors (TF-DEGs) and DEGs that were first-degree neighbors of the TF-DEGs (Supplemental Data Set S1h). Seven TFs with a significant nitrogen-specificity index (see Materials and methods) were identified (*P* < 0.05; Supplemental Data Set S1h; Figure S2). All of these except for *LBD19* have earlier been linked to nitrate responses in either *Populus* or *Arabidopsis* (references in Supplemental Data Set S4), demonstrating at least partial evolutionary conservation in nitrate responsiveness across these species.

### Cell expansion-related transcriptome responds rapidly to nitrate

To clarify the dynamicity of nitrate response in the different stages of vascular development, the DEGs were organized into “time bins” based on their first appearance during the time-course experiment. The genes in the “time bins” were grouped based on their log_2_(TPM+1)-transformed Transcripts Per Kilobase Million (TPM) values in the AspWood database (Sundell et al. 2017), which consists of high-resolution, spatial expression data in woody tissues of aspen (*P. tremula*) stem (Fig. 2e; Supplemental Data Set S1e). This analysis revealed the influence of the 2-h nitrate treatment predominantly on genes with preferential expression during xylem expansion (EX) according to the AspWood data (Fig. 2e; Figure S1b). At 4 h, the process of secondary cell wall formation appeared to be suppressed by nitrate based on the time bin analysis of the DEGs in the AspWood database (Fig. 2e; Figure S1b) as well as the enrichment of GO terms related to cell wall organization or biogenesis among the genes downregulated after 4 h of nitrate treatment (Fig. 2c). The 4-h time point also marked an increase in the numbers of time bin DEGs with the expression at a later stage of xylem maturation (CD) (Fig. 2e). Taken together, the results demonstrate that the 2-h time point captured the early effects of nitrate signaling especially on genes preferentially expressed during xylem cell expansion.

### Most xylem cell types respond to the nitrate signal

Single-cell RNA-sequencing (scRNA-seq) was performed to explore the nitrate-mediated regulation of xylem cell expansion on a cellular level in trees that were injected with KNO_3_ or KCl (control) and incubated for 2 h in the same way as for the time-course experiment. scRNA-seq libraries were prepared from protoplasts isolated from the surface of the woody tissue after peeling of the bark (Fig. 1, d to f). Single-cell RNA sequencing of 2 independent biological experiments yielded 13,067 and 11,454 cells for the control and nitrate-treated samples, respectively. Gene expression profiles were highly reproducible between the 2 experiments, with Pearson correlation coefficients of 0.89 and 0.91 for the control and nitrate-treated samples, respectively. Quality parameters for the scRNA-seq data for the experiment that was utilized for downstream analysis were assessed before and after pre-processing (Figure S3, a to e).

The transcriptome profiles from 20,566 cells were clustered using a graph-based approach, yielding 21 clusters (Fig. 3a). The quality metrics for each cluster are shown in Figure S3, f to h. Clusters were identified using both previously characterized marker genes (Fig. 3b) in *Populus* as well as genes enriched in different clusters based on the Wilcoxon test (Supplemental Data Set S3, a and b). References to literature for the cluster-specific genes in Fig. 3b are given in Supplemental Data Set S3b. We also performed transcriptomic correlations with existing gene expression datasets in woody tissues of *Populus*, including the high-resolution RNA-seq data from AspWood database (Sundell et al. 2017), spatial profiles (Du et al. 2023), single nuclei profiles (Conde et al. 2022; Schmidt et al. 2025) and laser capture microdissected (LCM) cells (Shi et al. 2017; Tung et al. 2023) to define our scRNA-seq cluster identities (Figure S4). Six promoter-GUS fusions were used to further validate identities of some of the clusters (Fig. 3e).

**Figure 3:**
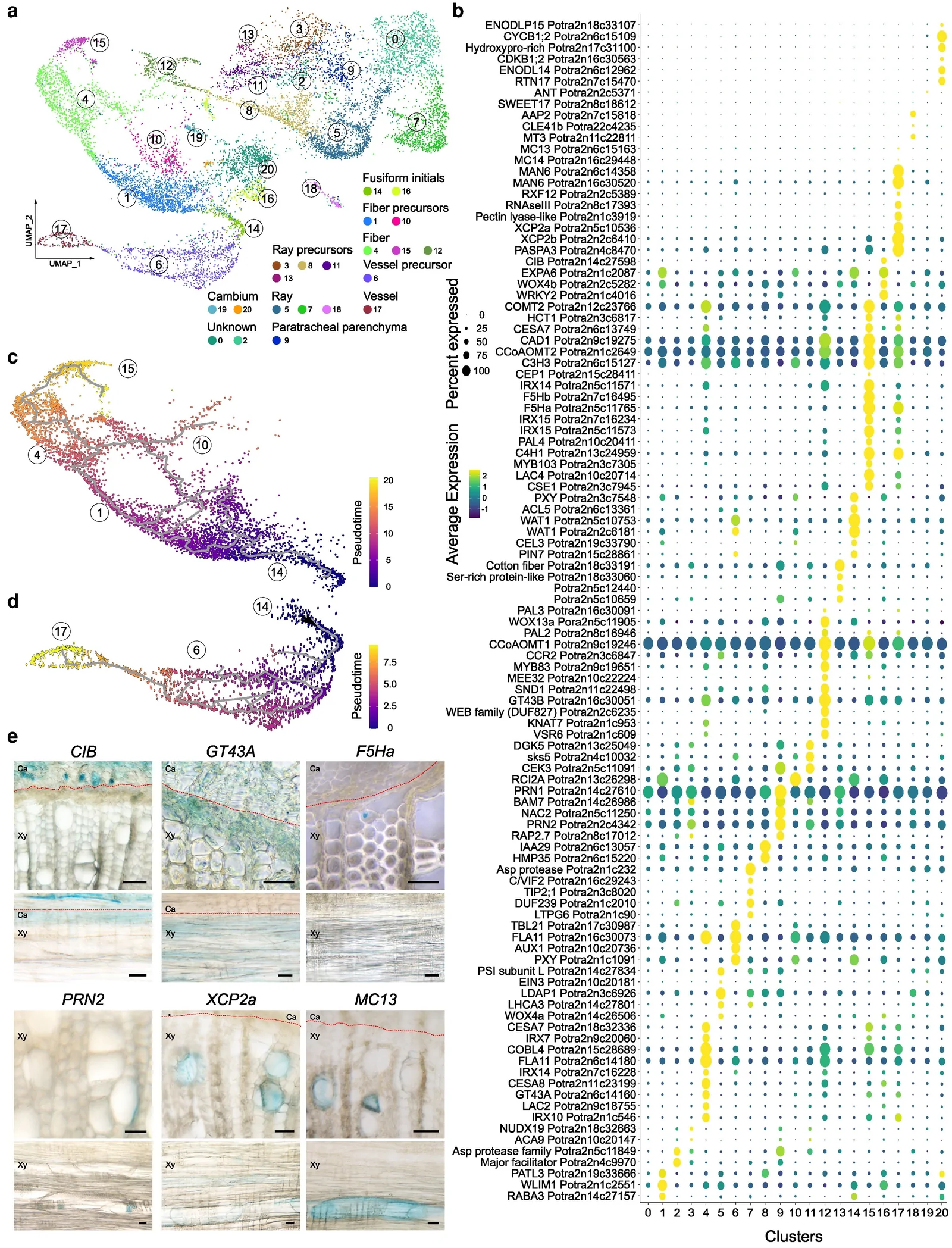
Single-cell transcriptome profiling in developing xylem of hybrid aspen. a) UMAP visualization of cell clusters in the scRNA-seq data. b) Marker genes enriched in each cluster. The genes were selected based on Wilcoxon test (Supplemental Data Set S3a) in Seurat and literature (Supplemental Data Set S3b). See Figure S4 for the correlation of the clusters to public transcriptomes. Gene annotations are based on the closest *Arabidopsis* homologs. c and d) Pseudotime developmental trajectory of xylem fibers and vessels showing differentiation from fusiform initials (cluster 14) to fibers (c) and vessels (d). e) Validation of cell cluster identity using promoter-GUS reporter lines. Transgenic hybrid aspen lines express GUS reporter gene under the control of *CRY2-INTERACTING BASIC HELIX-LOOP-HELIX PROTEIN* (*CIB*; Potra2n14c27598), *GLYCOSYLTRANSFERASE GT43A* (*GT43A*, Potra2n6c14160), *FERULATE 5-HYDROXYLASEa* (*F5Ha*, Potra2n5c11765), *PIRIN2* (*PRN2*, Potra2n2c4342), *XYLEM CYSTEINE PEPTIDASE 2a* (*XCP2a*, Potra2n5c10536), and *METACASPASE 13* (*MC13*, Potra2n6c15163) promoters. The results support the identities of fusiform initial cluster 16, marked by *CIB* promoter activity; fiber clusters 14 and 15, marked by promoter activities of *GT43A* and *F5Ha*, respectively; paratracheal parenchyma cluster 9, marked by *PRN2* promoter activity; and vessel cluster 17, marked by promoter activities of *XCP2a* and *MC13*. Ca, vascular cambium; Xy, xylem. Scale bar = 25 µm. The red dotted line depicts the boundary between cambium and expanding secondary xylem.

Briefly, clusters 5, 7, and 18 were annotated as rays based on the enrichment of photosynthesis-related genes and correlation with LCM data (Tung et al. 2023). Various secondary cell wall associated cellulose synthases (*CESA*) were among the highly expressed genes in clusters 4, 12, 15, and 17, xylan biosynthetic *IRREGULAR XYLEM* (*IRX9, IRX14, IRX15*) and glycosyltransferase *GT43A* genes in clusters 4 and 15, and core lignin-biosynthetic genes and laccases (*LAC*) in clusters 12, 15, and 17 (Fig. 3b). The transcriptomes of these 4 clusters (4, 12, 15, and 17) had the highest correlation to the AspWood sections from the zone of early xylem maturation (Figure S4, a and c), which suggests that these clusters are likely to contain vessels and fibers at different stages of differentiation. Correlation of the clusters 4, 12, and 15 with the LCM fiber transcriptomes (Figure S4, b and c) suggests that these 3 clusters contain fibers, while correlation of cluster 17 with the LCM vessel transcriptome and the expression of known vessel-specific genes, such as X*YLEM CYSTEINE PEPTIDASE* (*XCP2a* and *XCP2b*), ribonuclease (*RNAseIII*), and endo-beta-mannanase (*MAN6*), specifically in cluster 17 suggests that this cluster contains vessels (Fig. 3b). Clusters 14 and 16 were annotated as fusiform (vessel and fiber) initials, cluster 6 as vessel precursors, and clusters 1 and 10 as fiber precursors as they correlated with the early expanding samples of the AspWood database (Figure S4c). These annotations were supported by the analysis of enriched GO terms (Supplemental Data Set S3c) as well as developmental trajectories which demonstrated a bifurcated pseudotime backbone from the fusiform initial cluster 14 toward either the vessel (6 and 14) or the fiber (1, 10, 4, and 15) clusters (Fig. 3, c and d; Figure S6, a to f). Evidence for the presence of cambial cells was obtained through identification of 100 cells expressing the stem cell marker *AINTEGUMENTA* (*ANT*) in cluster 19 and cell division genes *CYCLIN* (*CYCB1;2*) and *Cyclin-dependent kinase* (*CDKB1;2*) in cluster 20 (Fig. 3b). Clusters 0, 2, 3, 8, 9, 11, and 13 did not clearly correlate with the earlier published datasets (Figure S4). However, transcriptome closeness to the ray clusters (Fig. 3a), correlation with the LCM ray sample and early AspWood cambial samples (Figure S4, b and c) suggested identity for clusters 3, 8, 11, and 13 as ray precursors. Cluster 9 showed high expression of *PIRIN2 (PRN2)* (Fig. 3b), which is specifically expressed in ray cells immediately adjacent to vessel elements (Fig. 3e), and was therefore annotated as paratracheal parenchyma.

### Nitrate regulates the expression of cell wall modifying proteins in the fusiform cells

Next, we investigated the nature of the cell types responding to the nitrate signal. First, a Canonical Correlation Analysis (CCA) analysis was used to integrate the scRNA-seq datasets generated from the control and nitrate-treated samples (Fig. 4a; Supplemental Data Set S3d). Clusters 0 and 2 did not show any correlation with the previous spatial or cellular *Populus* transcriptomes and were excluded from the analysis. Before preprocessing, 72.1% and 80.1% of reads from the control and nitrate-treated samples, respectively, mapped confidently to the transcriptome (Supplemental Data Set S3i). Pre-processing at cell and gene levels resulted in 11,514 and 9,052 cells with a median unique molecular identifier (UMI) count of 2,062 and 2,608, median gene count of 1,390 and 1,702, and median genes per UMI of 0.683 and 0.655 for control and nitrate-treated samples, respectively. A total of 28,217 unique genes were detected by scRNA-seq (27,180 and 26,721 in control and nitrate-treated samples, respectively), representing 95% of the genes detected by the bulk RNA-seq of the xylem tissues in the time-series experiment. Differential expression analysis between the nitrate-treated and control samples identified 1,446 DEGs (Supplemental Data Set S3e). The presence of DEGs in every cluster indicated that all xylem cell types were capable of sensing and responding to the nitrate signal (Figure S5). For instance, the early nitrate marker *NIR1* responded to the external nitrate supply in all xylem cell types, except for the small number of cells putatively involved in cell division in cluster 20 (Fig. 4b). However, nitrate responsiveness differed markedly among the xylem cell types (Fig. 4b; Figure S5). Differential enrichment of GO terms demonstrated that for instance *cell wall organization or biogenesis* was significantly upregulated in clusters 4 and 6, *nitrogen cycle metabolic process* was upregulated in cluster 18, and *cell cycle* was downregulated in cluster 4 (Fig. 4d; Supplemental Data Set S3f). The clusters containing the most nitrate responsive cell populations in the scRNA-seq data were the ray clusters 5, 7, 8, and 9 (Fig. 4c).

**Figure 4:**
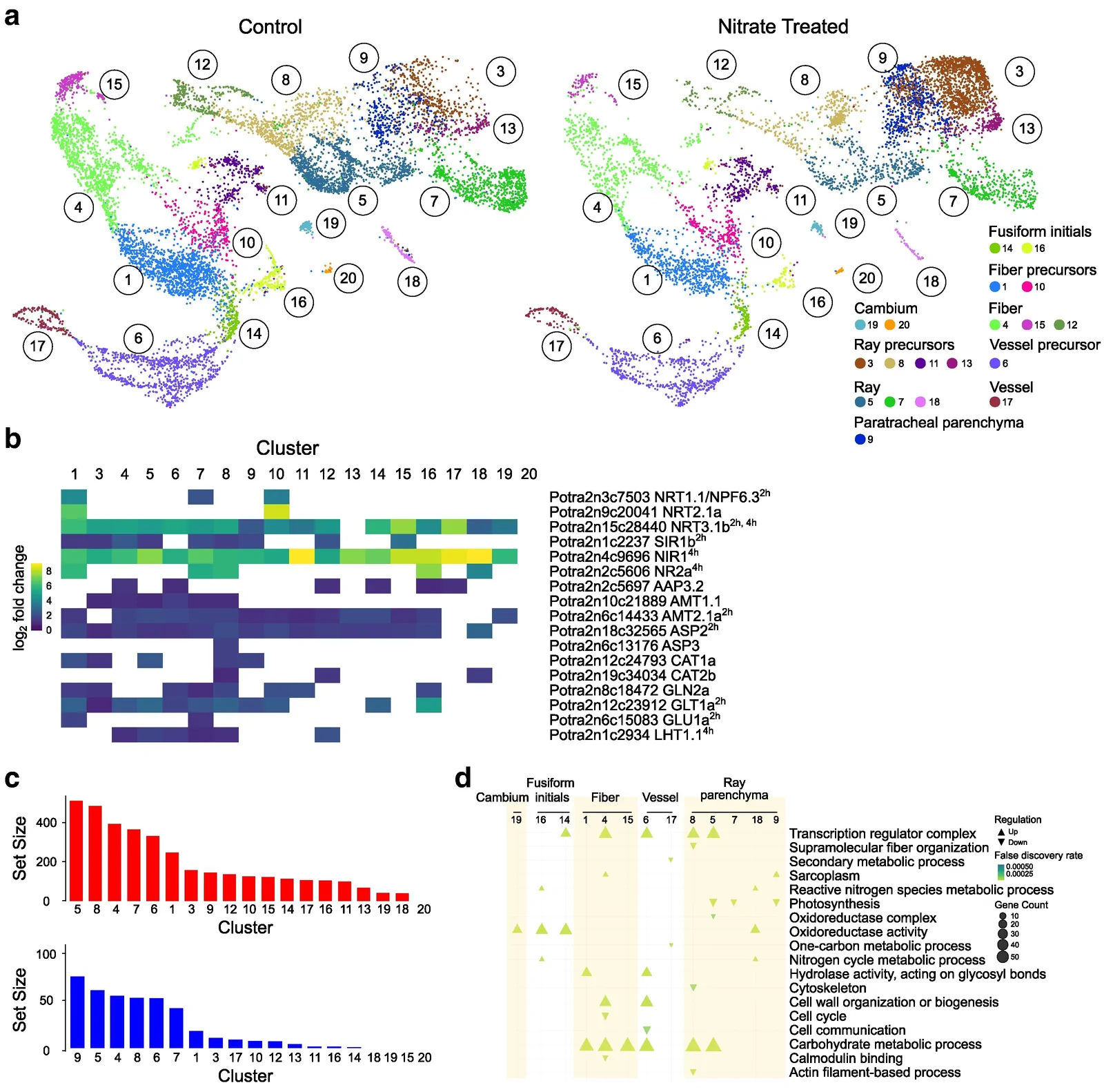
Gene expression analysis after nitrate treatment in different xylem cell types of hybrid aspen. a) UMAP visualization of the cells from control and nitrate-treated samples. Distinct colors depict unique cell clusters. Clusters 0 and 2 were excluded from this analysis. b) Cluster-wise expression profile of DEGs related to nitrogen uptake and metabolism in the scRNA-seq data. The superscript on the gene labels indicates that the gene was a DEG in the time-series study. *P. tremula* gene annotations are based on Zhang et al. (2025). c) Number of upregulated (upper panel) and downregulated (lower panel) genes identified in each cluster for nitrate versus control treatment. See Supplemental Data Set S3e for the list of DEGs in each cluster. d) Significant GO terms for the scRNA-Seq DEGs in the different xylem cell types. See details in Supplemental Data Set S3f.

In addition to rays, a strong response to nitrate was observed in fibers and vessels at the early stages of development (scRNA-seq clusters 1, 4, and 6) (Fig. 4c; Figure S5; Figure S6, g and h). In these cells, nitrate altered the expression of multiple genes belonging to the pectin methylesterase, pectin acetylesterase, xyloglucan endotransglucosylase/hydrolase, cellulose synthase, and expansin families (Supplemental Data Set S3e; Fig. 5a) that are all in different ways involved in xylem cell expansion (Scheible et al. 2004; Hacke et al. 2010; Plavcová and Hacke 2012). Interestingly, we found that 4 of the 5 lignin-biosynthetic genes were suppressed in the fiber and vessel clusters 4, 12, and 17 after nitrate application (Supplemental Data Set S3e; Fig. 5a). Analyses of cell wall chemistry in expanding xylem tissues of nitrate-treated trees corroborated these transcriptomic findings. Optical photothermal infrared (OPTIR) microspectroscopy revealed in transverse sections of the stem that a continuous, 72-h nitrate application significantly increased the level of methylesterified pectin in cell walls of xylem elements that were at an early phase of cell expansion, while the nonesterified pectin levels were less affected by nitrate compared to the control (Cohen's *d* = 0.968 vs 0.69, respectively; Fig. 5, b to d). Nitrate application induced also a slight delay in the onset of secondary cell wall lignification of the xylem elements (Fig. 5, e to g).

**Figure 5:**
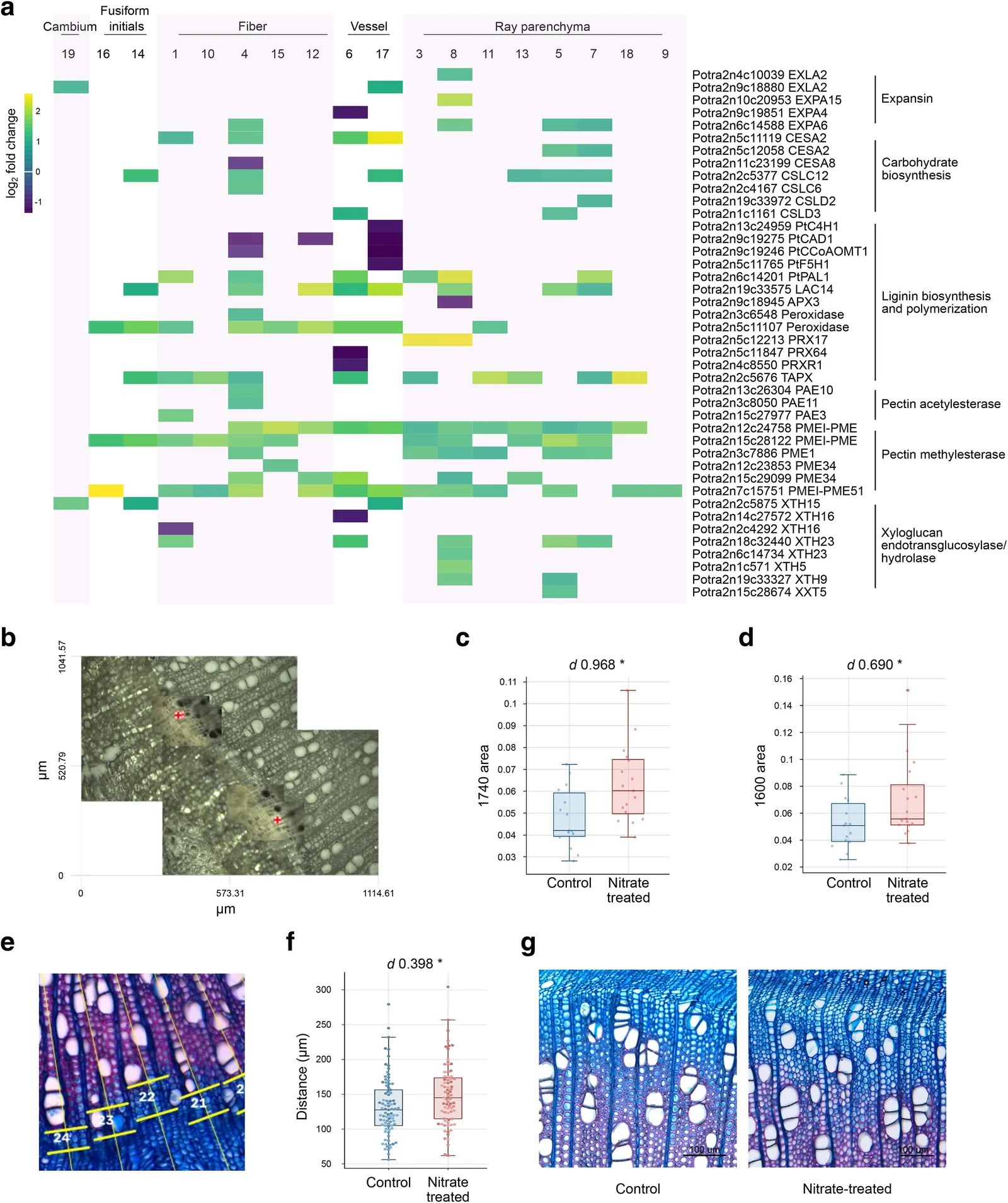
The effect of nitrate on cell wall remodeling genes and chemistry on a cellular level in hybrid aspen. a) Cluster-wise expression profile of DEGs related to cell wall biosynthesis and modification in the different xylem cell types in the scRNA-seq data. Gene annotations are based on the closest *Arabidopsis* homolog in TAIR. b to d) The effect of nitrate treatment on the level of pectin methylesterification. The cell wall chemistry of the most recent cambial derivate was analyzed in each file of the secondary xylem cells, as indicated by asterisks in (b), in transverse sections of stems by OPTIR microspectroscopy. *N* = 3. 15 to 19 spectra were collected for each treatment. c) The level of methylesterified pectin, estimated by the diagnostic IR vibration at 1,740 cm^−1^ as the area of the band in relation to the total area of the cell wall matrix. d) The level of nonesterified pectin, estimated by the diagnostic IR vibration at 1,600 cm^−1^ as the area of the band in relation to the total area of the cell wall matrix. While the *P*-value (Student's *t*-test) was below 0.05 (*) for both methylesterified and nonesterified pectin, Cohen's *d* values showed a large, highly meaningful effect (0.986) for the methylesterified pectin, and less for the nonesterified pectin (0.69). e to g) The effect of nitrate on the onset of lignification. e) The measurement of the onset of lignification. The onset was quantified by measuring the distance from the last cambial cell to the first lignified cells, identified by the appearance of the pink-stained cell walls in transverse sections stained with safranin–alcian blue. The image shows an example of a measurement. *N* = 3. At least 25 measurements were done in each of 3 sections from each sample. f) Quantification of the onset of lignification in response to treatment with nitrate or control. While the *P*-value (Student's *t*-test) was below 0.05 (*), Cohen's *d* value indicates only small-to-medium effect size (0.398). g) Transverse sections from the secondary xylem tissues of control and nitrate-treated trees. The transverse sections for (b to g) were taken from trees that were grown in peat-based soil with an optimal fertilization regime corresponding to a total amount of ∼1 g nitrogen for 7 wk and depleted for nitrogen during the 4 wk before continuous, 72-h injection of 5 mM KNO_3_ or 5 mM KCl (as control) into the base of the stem. Sections were taken 3 cm above the injection point.

### CYTOKININ RESPONSE FACTOR 4 mediates nitrate-stimulated xylem cell expansion

To identify transcriptional regulators of nitrate-mediated xylem cell expansion, we examined TFs that responded to nitrate in the different xylem cell types. Out of these, *WRKY40, ASR3*, *ATAF1*, *HHO5*, and *LBD38* are known nitrate-responders (see references and gene models in Supplemental Data Set S4). Novel types of TFs in nitrate signaling included *ERF1B*, *ERF4*, *ERF110*, *HB-8*, *FBH4*, *HAT22*, bZIP TF, *NERD*, *WRKY48*, and *CBF1*. We further refined our analyses to identify TF-DEGs associated with xylem expansion based on 3 criteria: (i) upregulation in response to nitrate in the scRNA-seq data, (ii) preferential expression in the expanding xylem according to the AspWood dataset, and (iii) expression within the fusiform developmental trajectories encompassing clusters 1, 4, 6, 10, 15, and 17 (Fig. 6, a to c). There were 14 TF genes fulfilling these criteria (Fig. 6, b and c). Among these, we identified the *Populus* transcription factor *CYTOKININ RESPONSE FACTOR 4* (*CRF4*, Potra2n15c29002, aka *ETHYLENE RESPONSE FACTOR 85*) that was already previously linked to xylem development (Seyfferth et al. 2021). *CRF4* expression increased in response to nitrate in all cell clusters, but significantly in clusters corresponding to fiber precursors (1), fibers (4), vessel precursors (6), and rays (5, 7, 8) (Fig. 6d). Taken together, the results suggest a function for CRF4 in xylem cell expansion. Furthermore, *CRF4* was co-expressed with *NRT1.1/NPF6.3*, *DEFECTIVE REGION OF POLLEN 1 (LRL1)*, and *ETHYLENE RESPONSE FACTOR 4 (ERF4)* in both fibers and vessels (Fig. 6e; Figure S7; Supplemental Data Set S1i), consistent with a shared role in regulating nitrate-dependent xylem development.

**Figure 6:**
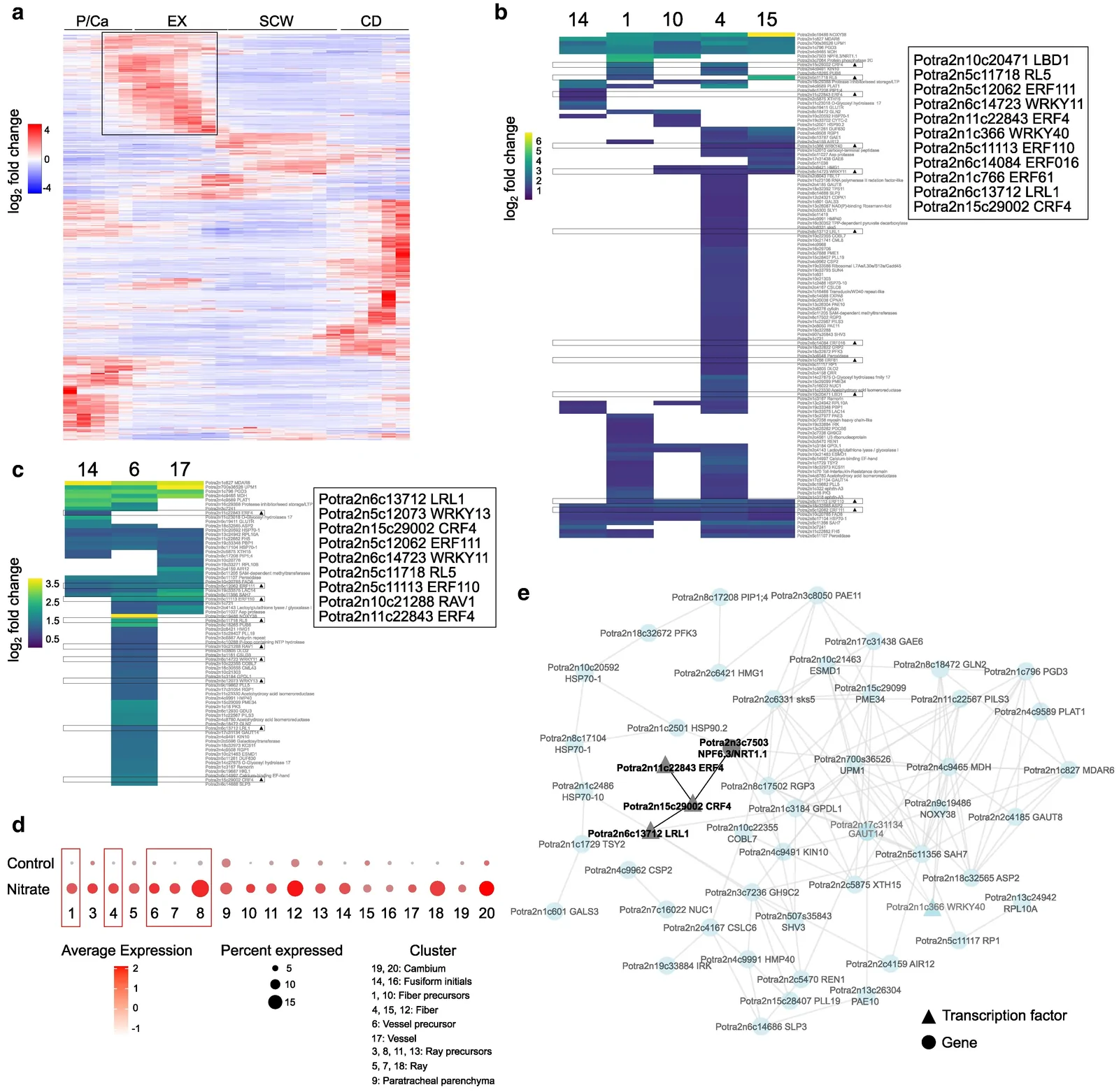
Gene expression circuits in the vessel and fiber trajectories. a) Heatmap of significantly upregulated genes from scRNA-seq data according to their log_2_(TPM+1)-transformed expression values (scaled for each gene) during cambial growth and wood development mapped to Tree1 in the AspWood database. P/Ca, phloem/cambium; EX, expanding xylem; SCW, secondary cell wall formation; CD, cell death. The box marks the expression of genes with comparatively high expression in the expanding xylem region. The list of the DEGs can be found in Supplemental Data Set S3e. b and c) The average expression of DEGs from the scRNA-seq data in the fiber trajectory of clusters 14, 1, 10, 4, and 15 (b) and the vessel trajectory of clusters 14, 6, and 17 (c) in control versus nitrate treatment, respectively. Transcription factor DEGs are shown within the box. For complete list of the DEGs in this heatmap, see Supplemental Data Set S3h. d) Cluster-wise expression of *CRF4* after nitrate treatment. The boxes show clusters where expression changes are significant (absolute log_2_ fold change >1 and FDR < 0.01). e) A subnetwork of fiber-cell expansion. The subnetwork was extracted from the whole-transcriptome co-expression network for TF-DEGs and their first-degree DEG neighbors that were specifically expressed in the phase of xylem expansion in the AspWood database and present in the scRNAseq fiber trajectory. Genes co-expressed with *CRF4* are shown in bold text.

To investigate if *CRF4* is involved in nitrate-mediated cell expansion, we analyzed the growth parameters and xylem cell size in transgenic hybrid aspen plants overexpressing the *P. tremula CRF4*. Overexpression of *CRF4* increased diameter of the fibers and showed a trend toward increased diameter of the vessel elements (Figure S8), therefore mimicking the stimulatory effect of nitrate on xylem cell expansion (Fig. 1g). Next, we created CRISPR-Cas9 lines in transgenic hybrid aspen trees targeting both *CRF4* and its paralogous gene *CRF3* (Potra2n12c23983). Three different lines were identified for 2 different CRISPR-Cas9 constructs as full knockout lines for both *CRF3* and *CRF4* (Table S2). All 6 lines grew normally except for line 43 that was smaller and line 35 that had slightly smaller stem diameter (Figure S9). The 2 lines, 35 and 43, displayed also smaller sizes of the xylem elements in transverse sections of the stem even though it was statistically significant only for line 43 (Figure S9). Lines 35 and 43, which showed the greatest deviations from the wild type, were selected for further analyses. Although the growth of line 43 differed significantly from that of line 35, both lines exhibited similar secondary xylem anatomy (Fig. 7a), a similar tendency to accumulate more nitrate in the xylem sap than the wild type following nitrate treatment (Figure S10a), and similar transcriptional responses (Supplemental Data Set S2h), providing the clearest opportunity to assess the functional consequences of CRF3/CRF4 loss. Next, we tested the effect of nitrate on cell expansion in the wild type and lines 35 and 43 by measuring the size of newly formed xylem elements in protoplasts isolated from the expanding xylem of trees treated with nitrate for 3 d (Fig. 7b; Figure S10b). In wild-type trees, application of nitrate significantly increased the protoplast size, with an effect size of *r* = 0.217 (Fig. 7b). In contrast, the CRISPR-Cas9 lines exhibited very weak to negligible increase in protoplast size following nitrate treatment (*r* = 0.022 and 0.097, respectively), indicating that their expanding xylem elements were impaired in sensing or responding to nitrate. Together, these results highlight the roles of the 2 CRFs in mediating nitrate-induced xylem cell expansion, revealing a mechanistic link between nitrate sensing and vascular development.

**Figure 7:**
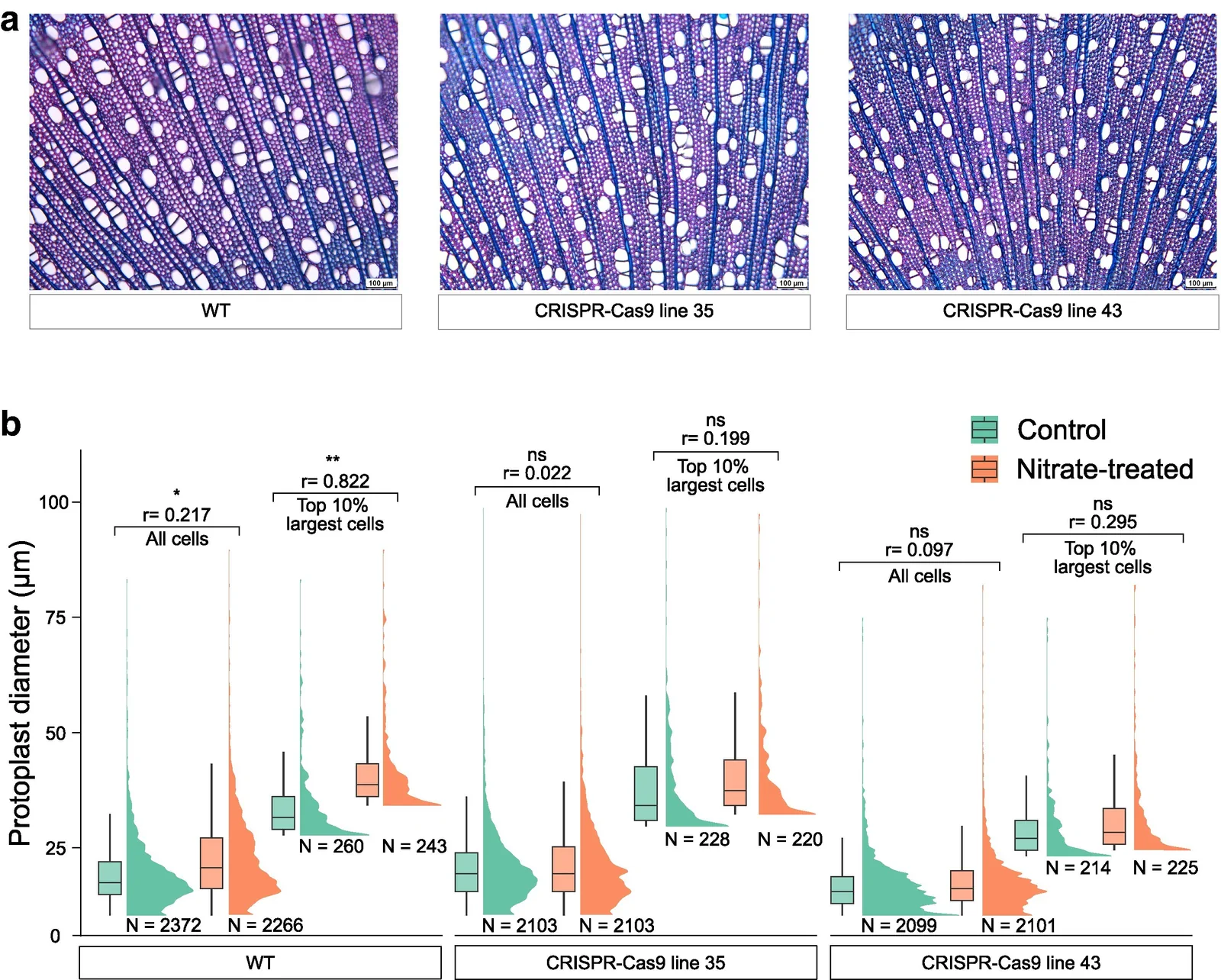
CRF4 together with CRF3 are required for xylem cell expansion in response to nitrate signal in hybrid aspen stem. a) Wood anatomy in 2 CRISPR-Cas9 lines targeting hybrid aspen *CRF4* and *CRF3* and in wild-type trees that were grown for 11 wk in the greenhouse. Representative images of wood are shown in transverse sections taken from the base of the stem. Scale bar = 100 µm. b) Cell sizes in developing xylem of stem tissues in wild type and the 2 CRISPR-Cas9 lines in response to nitrate treatment. Trees were grown in peat-based soil with an optimal fertilization regime corresponding to a total amount of ∼1 g nitrogen for 7 wk and depleted for nitrogen during 4 wk before nitrate treatment. 5 mM KNO_3_ or 5 mM KCl (as control) was continuously injected into the base of the stem. The cell sizes were quantified from protoplasts isolated from developing stem tissues 72 h after treatment and measured for the diameter. *N* = number of protoplasts from the developing xylem taken for size measurements from 2 to 3 individual trees per treatment for each line. Statistics were performed for all cells as well as the largest 10% of the protoplast population (shown alongside all cells) within each genotype and treatment. Differences in cell sizes were evaluated using a linear mixed-effects model. Estimated marginal means are shown with 95% confidence intervals. Significance levels are shown as ns (not significant), with 1 asterisk (*P*  *<* 0.05) or 2 asterisks (*P*  *<* 0.01). The magnitude of nitrate-induced effects on cell expansion was quantified using rank-biserial correlation (*r*) calculated from Wilcoxon–Mann–Whitney comparisons. For explanation of the statistics, see the legend in Fig. 1.

## Discussion

### Response to nitrate in developing xylem is short-lived

Nitrate rapidly induced transcriptomic changes in the developing xylem tissue of hybrid aspen trees (Fig. 2), demonstrating not only uptake of nitrate along its transport route but also sensing of nitrate to coordinate xylem development. Based on these findings, we propose that developing xylem continuously monitors the levels of nitrate in the transpiration stream and rapidly adjusts cellular processes, such as cell expansion, in response to the levels of nitrate in xylem sap. However, the transcriptional response of developing xylem to nitrate was rapidly attenuated, even when nitrate was continuously supplied through the transpiration stream (Fig. 2; Supplemental Data Set S2c). These observations suggest that developing xylem responds primarily to transient changes in nitrate availability rather than to sustained nitrate supply. Consequently, nitrate signaling is unlikely to durably reprogram xylem developmental programs or xylem function. In contrast, the long-term changes in wood formation observed following nitrate fertilization (Renström et al. 2024) are more likely to reflect the nutritional effects of prolonged nitrogen availability than persistent activation of nitrate signaling.

The transient nature of the nitrate response is consistent with similar observations in *Arabidopsis* roots (Krouk et al. 2010; Undurraga et al. 2017; Contreras-López et al. 2022) and with the finding that the master regulator NLP7 binds many of its targets only transiently (Alvarez et al. 2020). The fact that our experiments were performed in developing xylem rather than roots demonstrates that transient nitrate signaling is not confined to tissues taking up nitrate but may represent a general principle of nitrate sensing throughout the plant.

### Developing xylem cells respond differently to nitrate

Cellular profiling is crucial for elucidating the regulatory networks that underlie cell-specific responses to nitrogen (Contreras-López et al. 2022). Our scRNA-seq analysis revealed that all xylem cell types, spanning multiple developmental stages as well as a subset of cambial cells, responded to nitrate. However, the magnitude of the transcriptional response varied substantially among cell types, with the highest numbers of DEGs observed in clusters corresponding to precursors of vessels and fibers (Fig. 4c).

Another cell type with a strong and fast response to nitrate was the rays (Fig. 4c), suggesting that nitrate is being taken up from the xylem sap into the radial transport route of the rays. Such transport in the radial dimension of the stem has not been experimentally demonstrated but indirect evidence exists on the basis of altered nitrate allocation in the inflorescence stem of *Arabidopsis* mutants for nitrate transporter family members *NRT1.8* (Li et al. 2010) and *NRT1.5* (Lin et al. 2008). Our data on nitrate-induced expression of several members of the nitrate transporter family in xylem rays supports radial transport of nitrate in the stem and provides candidates for facilitating such transport.

In addition to the magnitude of responsiveness, the different cell types displayed differences in their transcriptomes in response to nitrate. In rays, a dominant response, according to the GO terms, was suppression of photosynthesis (Fig. 4d). Similar response was earlier reported in young internodes of *P. trichocarpa* stem (Euring et al. 2014). It is currently difficult to understand the significance of this response, especially since nitrogen is well known to improve photosynthetic efficiency in leaves. Several studies have, however, reported that nitrogen fertilization beyond optimal levels can suppress photosynthetic performance (Sperling et al. 2019; Waring et al. 2023), but the nitrate levels that were applied in this study were kept within a physiological range that prevails in the xylem sap (Hu and Guy 2020; Figure S10a). In any case, our results provide support for nitrate having a role in diverting metabolic fluxes between carbohydrate biosynthesis and photosynthesis across the developing xylem tissues. This response, likely related to the short-term signaling function of nitrate, illustrates the kind of responses that can be hidden when analyzing the function of nitrogenous compounds in the long-term experiments with bulk tissues.

### Xylem cell expansion is rapidly modified by nitrate

The transcriptomic responses in the early developing xylem elements suggest involvement of nitrate signaling in xylem cell expansion (Fig. 2; Fig. 6a), consistent with the rapid, stimulatory effect of nitrate on xylem cell expansion observed in this study (Fig. 1g). A question arises whether nitrate stimulates xylem cell expansion directly or indirectly by modulating the processes of cell division or secondary cell wall formation. In *Arabidopsis*, nitrate stimulates organ size through both cell expansion and division (Moreno et al. 2020; Abualia et al. 2022; Pereyra et al. 2023). We observed a significant enrichment of GO terms related to *cell cycle* in genes downregulated in response to nitrate (Supplemental Data Set S3f; Fig. 4d), suggesting that nitrate might regulate xylem cell expansion by suppressing cambial cell division and thereby promote transition to xylem expansion. However, several lines of evidence pointed to a direct, cell-autonomous role of nitrate on xylem cell expansion. First, upregulation of the primary nitrate responsive genes demonstrates nitrate uptake and responsiveness in the early developing xylem elements (Fig. 4b). Second, vessels and fibers at the earliest stages of development showed overrepresentation of GO terms that were related to cell wall modification and carbohydrate biosynthesis (Fig. 4d). In these cells, nitrate upregulated the expression of several genes in families, such as XTH, EXP, PAE, and PME (Fig. 5a), known to be central in cell wall biosynthesis and modification during xylem cell expansion (Siedlecka et al. 2008; Nishikubo et al. 2011). Such changes are likely to underlie the stimulatory effect of nitrate in pectin methylesterification, identified in our study (Fig. 5, b to d) as well as by others (Nunez-Pascual et al. 2025). Nitrate did not, however, influence carbohydrate metabolism as strongly at later stages of xylem maturation; instead it suppressed the expression of lignin-biosynthetic genes (Fig. 5a). A trend toward a delay in the onset of lignification was also observed in response to nitrate (Fig. 5, e to g). Our results therefore suggest that nitrate regulates xylem cell expansion both by stimulating the biosynthesis and modification of the primary cell walls and by suppressing the secondary cell wall biosynthesis, in particular lignification.

### CRF4 is involved in nitrate-mediated xylem cell expansion

Although substantial information exists regarding the molecular machinery that drives cell expansion, alternative pathways are likely to mediate nitrate-induced expansion in specific cell types, such as xylem elements (Pereyra et al. 2023). We therefore examined transcription factors induced by nitrate in developing xylem and identified several regulators previously implicated in nitrate responses in other species, together with numerous transcription factors not previously associated with nitrate (Figure S2; Supplemental Data Set S4). Among the candidate regulators of nitrate-induced xylem cell expansion, we identified the transcription factor *CRF4*, which has previously been implicated in the regulation of xylem development in hybrid aspen (Seyfferth et al. 2021). Interestingly, the *Populus CRF4* is a homolog of *Arabidopsis CRF4*, which was earlier identified as a central transcriptional hub in nitrate signaling (Varala et al. 2018). Our functional studies finally revealed a critical function for CRF4 together with CRF3 in nitrate-mediated xylem cell expansion in hybrid aspen (Fig. 7; Figure S9). This function was not evident in the knockout lines for *CRF3* and *CRF4* continuously fertilized with nitrogen (Figure S9), suggesting that CRF4 together with CRF3 mediates the transient local nitrate signal rather than the longer-term nutritional effects of root-supplied nitrate. This CRF-dependent pathway may be particularly important when nitrate availability around developing xylem changes rapidly, allowing xylem expansion to be transiently adjusted to local conditions. However, the signaling pathway linking CRF3/CRF4 activity to the cellular processes that drive xylem cell expansion remains unknown. Interestingly, *CRF4* was co-expressed with *ERF4* (Fig. 6e), a transcription factor previously implicated in the regulation of cell size in *Arabidopsis* leaves through endoreduplication (Ding et al. 2021, 2022). Endoreplication has been implicated in protoxylem development in *Arabidopsis* primary roots (Wendrich et al. 2020), but it has not been observed in the secondary xylem of *Populus* and is generally considered to be absent during wood formation (Kondorosi et al. 2000; Ye et al. 2014). ERF4 has also been shown to regulate demethylesterification of homogalacturonan, and it is tempting to speculate, based on our results, that ERF4 together with CRF4 promotes xylem cell expansion by controlling the degree of pectin methylesterification.

Together, our results identify a transient transcriptional response to nitrate within the wood-forming region and establish CRF4 as a component linking nitrate signaling to xylem cell expansion in hybrid aspen trees. An important next step is to determine how nitrate signaling is coordinated across the 3-dimensional organization of developing stems. Combining spatial transcriptomics, nitrate biosensors, and cell-type-specific perturbations should clarify the extent to which nitrate responses are cell autonomous or coordinated across neighboring tissues and developmental gradients. Such studies will provide a broader framework for understanding how trees integrate fluctuating nitrate availability with wood development.

## Materials and methods

### Plant material and growth

Hybrid aspen (*Populus tremula* L. × *P. tremuloides*, Michx.) clone T89 rooted cuttings were used for all experiments. Growth conditions and fertilization regimes are indicated for each experiment separately.

Transgenic promoter-GUS fusions were created for 3 genes, *CRY2-INTERACTING BASIC HELIX-LOOP-HELIX PROTEIN* (*CIB*; Potra2n14c27598), *XCP2a* (Potra2n5c10536), and *FERULATE 5-HYDROXYLASE* (*F5Ha*, Potra2n5c11765), by amplifying 2,418-, 2,068-, and 1,551-bp long promoter fragments, respectively, from hybrid aspen genomic DNA (for primers, see Table S1). The promoter fragments were cloned by GATEWAY cloning into pKGWFS7 and transformed into hybrid aspen clone T89 (Nilsson et al. 1992). Earlier reported promoter-GUS fusions for *PRN2* (Zhang et al. 2020), *MC13* (Bollhöner et al. 2018), and *GT43A* (Ratke et al. 2015) were also utilized. Two moderately expressing lines of each construct with 2 to 3 biological replicates were planted in a greenhouse (18:6 h day/night cycle) and analyzed for the localization of β-glucuronidase (GUS) activity. Radial and longitudinal sections (55 to 70 µm) were observed under a Leica DMI8 microscope with a Leica DFC7000T color camera or a Leica M205FCA microscope with a Leica K5C-700010242537 color camera and LasX software (Leica Microsystems, Wetzlar, Germany).

To generate CRISPR-Cas9 lines for *CRF4* (Potra2n15c29002) and *CRF3* (Potra2n12c23983), guide RNAs targeting 2 selected sequences were designed using Cas-Designer (http://www.rgenome.net/cas-designer/) (Table S2). Three constructs were generated. Two of them, resulting in lines 7, 10, and 35, were generated following an earlier protocol (Xing et al. 2014). Fragments for cloning were amplified from pCBC-DT1T2 using the oligo pairs G8 Forward and G10 Reverse for lines 7 and 10, and G1 Forward and G7 Reverse for line 35 (Table S1). The 15 μL reaction volume contained 100 ng vector, 100 ng insert fragment, 1× T4 ligase buffer, 30 U T4 DNA ligase (Thermo Scientific), 0.1 mg/mL bovine serum albumin (BSA), and 1 μL of Eco31I, and was subjected to 50 thermal cycles (37 °C 5 min; 16 °C 5 min) followed by heat inactivation and transformation into *Escherichia coli* strain DH5α. Amplified fragments purified from agarose gel were cloned into the pKSE401 binary vector and introduced into *Agrobacterium* strain GV3101pMP90 (Koncz and Schell 1986). For the third construct, resulting in lines 17, 35′, and 43, G3 Forward and G6 Reverse primers (Table S1) were used with GreenGate assembly method (Lampropoulos et al. 2013). All 3 constructs were transformed into hybrid aspen clone T89 as described before (Nilsson et al. 1992). Transformed lines were screened for mutations by PCR amplification of sequences flanking the targeted regions using genotyping primers (Table S1). Amplified fragments were gel-purified and sequenced using reverse primers. Sequencing results were analyzed using the CLC Main Workbench v23, and indels in the CRISPR-Cas9 lines were identified (Table S2).

### Protoplast isolation

Protoplasts were isolated for measurement of the sizes of the developing xylem cells as well as for the single-cell RNA-seq experiment according to an earlier protocol (Lin et al. 2014) with modifications. Briefly, stem pieces were debarked and subjected to cell wall hydrolysis with a solution containing 1.5% (wt/vol) cellulase R-10, 0.4% (wt/vol) macerozyme R-10, 0.5 M mannitol, 20 mM KCl, 10 mM calcium chloride (CaCl_2_), 20 mM 4-morpholineethanesulfonic acid (MES; pH 5.7), and 0.1% (wt/vol) BSA for 2 h in dark at room temperature. The digested pieces were then transferred to wash solution (5 mM glucose, 125 mM CaCl_2_, 154 mM NaCl, 4 mM MES, and 5 mM KCl) on ice and gently inverted for 1 to 2 min to allow the release of protoplasts from digested tissue. The protoplasts were washed thrice with wash solution using Allegra X-I5R centrifuge (Beckman Coulter) at 100 × *g* (at an acceleration of 8 and deceleration of 4) for 5 min at 4 °C and finally resuspended in 500 µL of solution containing 0.5 M mannitol, 20 mM KCl, 20 mM MES, and 0.1% BSA.

### Nitrate application for the RNA-seq experiments

Hybrid aspen trees (clone T89) were grown for 14 wk in the greenhouse, first for 12 wk under low nitrogen conditions followed by 2 wk of nitrogen starvation prior to nitrate applications. Trees were grown in peat-based substrate with low nitrogen content (Solmull, Hasselfors Garden, Sweden). The substrate was pretreated with 2 g lime per liter of peat to increase pH to approximately 5.0 to 6.0. Greenhouse and light conditions were kept the same as in Renström et al. (2024). Trees were treated with 50 mL RIKA-S (SW HORTO AB) based fertilizer once a week. Nitrogen fertilization started with 6 mM ammonium nitrate (NH_4_NO_3_) during the first week and was gradually increased to 40 mM NH_4_NO_3_ by the final week, resulting in a total of 0.38 mg of nitrate supplied to the trees by the final week 12 (Fig. 1a). After 12 wk, leaves were removed from the lowest part of stem, and fertilization was withheld for the following 2 wk. After the nitrogen-depletion period, 2 mL 5 mM KNO_3_ or KCl (as control) was injected into the xylem sap at the base of the stem using two 1-mL syringes (Injekt-F Solo) that were connected to 1.2 mm Ø Sterican needles, as adapted from previous protocols (Sano et al. 2005; Umebayashi et al. 2007). A total of 0.14 mg of nitrogen was injected into each tree. The piston of the syringe was removed to allow the solution to be drawn out of the syringe by the tension of the xylem sap, and a drop of the solution was placed on the stem to prevent air from entering the needle. The 2 syringes were placed on opposite sides of the stem (Fig. 1b). Samples were collected from 4 biological replicates for each treatment (KNO_3_ or KCl) 0, 2, 4, 8, 12, 24, and 48 h after the injections. Stem pieces were collected at the height of 40 to 60 cm from the injection point and frozen in liquid nitrogen for the time-series RNA-seq analysis, and at the height of 20 to 60 cm for the scRNA-seq experiment.

The efficiency of the injection method was evaluated by following the fate of a stain in parallel experiments. 0.375% (wt/wt) acid fuchsin was injected into the base of hybrid aspen trees following the same procedure as for the KNO_3_ and KCl injections. Two hours after injection, stem segments were collected at 10-cm intervals up to 60 cm from the injection point from 6 replicate trees (Fig. 1c). Hand sections were taken and imaged by a stereomicroscope Leica M205FA (Leica Microsystems, Wetzlar, Germany). The positively stained area was quantified as a percentage of the total wood area in a transverse section of the stem using ImageJ.

### Bulk RNA-seq of developing xylem tissue and differential gene expression analysis

#### RNA extraction

A 10 cm frozen stem piece, collected 40 to 60 cm from the injection point, was debarked. The developing xylem was scraped from the surface of woody tissue with a scalpel and homogenized to a fine powder. RNA was extracted using a combination of the CTAB method (Chang et al. 1993) and RNeasy Plant Mini kit (QIAGEN). Briefly, 800 µL of prewarmed (65 °C) CTAB solution (2% cetyltrimethylammonium bromide [CTAB], 2% polyvinylpyrrolidone, 100 mM Tris-HCl [pH 8.0], 25 mM EDTA, 2.0 M sodium chloride [NaCl], 18 µL of β-mercaptoethanol) was added to 100 mg of frozen sample, vortexed for 25 s and incubated at 65 °C for 1 min. Samples were extracted with 800 µL of chloroform-isoamylalcohol (24:1) twice by mixing gently and centrifuged at 3,000 rpm for 10 min at room temperature. RNA was precipitated using 2.5 M lithium chloride (LiCl) from the upper phase by keeping on ice for 1 h and centrifugation at 13,000 rpm for 20 min at 4 °C. The pellet was dissolved carefully in 400 µL of RLT buffer (QIAGEN). RNA was extracted using RNeasy Plant Mini kit as per the manufacturer's instructions except that an on-column DNase I digestion (Invitrogen) was performed after the first wash with 350 µL of RW1 buffer. Finally, RNA was eluted twice with 33 µL of RNase-free milli-Q water. RNA was quantified with a Nanodrop 1000 Spectrophotometer (Thermo Scientific), and the quality was assessed with an Agilent 2100 Bioanalyzer (Agilent Technologies, Santa Clara, CA). The RIN values ranged from 8.6 to 9.7.

#### RNA-sequencing

Fifty-two samples, including 4 replicates at each of the 7 time points (0, 2, 4, 8, 12, 24, and 48 h) after nitrate and control treatments, were sequenced on the Illumina (San Diego, USA) NovaSeq X platform with paired-end setup (300 bp). RNA-seq data analysis was performed using nf-core pipeline (Ewels et al. 2020). Briefly, raw reads were trimmed to remove low-quality bases (Quality Phred score <20) and sequencing adapters using Trim Galore v0.6.7. The preprocessed reads were quantified with Salmon v1.10.0 using the transcriptome index of *P. tremula* v2.2.

#### Differential gene expression

Differential gene expression analysis between nitrate and control treatments was done using DESeq2 v1.44.0. DEGs, fulfilling the selection criteria of log_2_ fold change (LFC) > 1 and false discovery rate (FDR) < 0.05, were reported significant. The variance-stabilized (VST) read counts were utilized for generating heatmaps with gplots v3.1.3.1. The absolute TPM values, GO enrichments, and the data for the gene co-expression networks were obtained from https://plantgenie.org/ (Sundell et al. 2015). Genes having VST expression values in at least 2 biological replicates at each time point were taken as a background gene set for GO enrichment analysis. The *k*-means clustering was performed on the log_2_(TPM+1)-transformed expression data with the hclust function of R v4.2.2 assuming a number of clusters (*k*) as 10, and visualized with ggplot2 v3.5.1.

#### Gene co-expression network

Seidr v0.14.2 was used to compute different network inference algorithms, including ARACNE, CLR, GENIE3, linear SVM (LLR- ensemble), mutual information, Narromi, partial correlation, Pearson correlation, Spearman, TIGRESS, and partial least squares feature selection method (Schiffthaler et al. 2023) on all genes having a VST expression value. Inverse rank product was calculated to aggregate the networks obtained from different methods, and the edges were filtered with a noise-corrected backbone at a sigma of 1.34. Network clustering was performed following Leiden algorithm using igraph v2.1.4 and visualized in Cytoscape v3.10.3.

*Populus* transcription factor (TF) genes (extracted from https://plantgenie.org) were used to construct a subnetwork containing all TFs and their first-degree DEG neighbors. The nitrogen-specificity index for the differentially expressed TFs (TF-DEGs) was calculated as reported earlier (Varala et al. 2018). First, the best *Arabidopsis* homologs were identified for all DEGs present in the subnetwork. Genome-wide transcription factor binding profiles were retrieved for *Arabidopsis* homologs of 11 out of the 60 TF-DEGs identified in our study from the *Arabidopsis* DAP-seq database (O’Malley et al. 2016; Ecker 2017), and the corresponding target genes were compared with the DEGs identified in our study. TF-DEGs with significantly higher number of targets among the DEGs relative to their genome-wide distribution, tested using 1-tailed Fisher test (*P* < 0.05), were considered specific to nitrate response (Supplemental Data Set S1h).

A subnetwork for fiber-cell expansion was extracted from the whole-transcriptome co-expression network for genes that were upregulated in the scRNA-seq fiber trajectories (clusters 1, 4, 10, 14, and 15) and specifically expressed during xylem expansion phase according to the AspWood dataset.

### scRNA-seq and differential gene expression analysis

#### Protoplast isolation and scRNA-seq

After 2 h of nitrate treatment, stem pieces were harvested 20 to 60 cm above the injection point for scRNA-seq. The scRNA-seq experiment was performed twice, but only the dataset with the higher cell recovery was used for downstream analyses. One experiment recovered approximately 3,955 cells, whereas the other yielded approximately 20,000 cells, providing greater coverage and resolution for subsequent analyses. To account for the inherent variation between individual plants, 5 plants for each treatment (control and nitrate-treated) were harvested for protoplast isolation and pooled for scRNA-seq. Protoplasts were isolated as described above, except that the suspension was filtered with a 40-μm cell strainer. The concentration and viability were estimated by trypan blue staining.

The high-throughput scRNA-seq was performed using the 10× Genomics Chromium technology. The sequencing libraries were prepared according to the Chromium Next GEM Single Cell 3ʹ Reagent Kits v3.1 User Guide CG000315 (rev E) for targeting 10,000 cells. The quality of DNA libraries was analyzed using an Agilent 2100 Bioanalyzer, and paired-end reads were generated on an Illumina NovaSeq 6000 SP-100 with a custom read setup of 28-10-10-90.

#### Clustering and annotation of the cell types

The raw scRNA-seq reads were aligned to the *P. tremula* reference genome v2.2 using the 10× Genomics Cell Ranger v7.0.0 pipeline (Zheng et al. 2017) that also performs barcode processing and cell calling with the intron mode option. The resulting expression matrices were processed with the Read10X_h5() function in Seurat v4.3.0.1 (Hao et al. 2021) to return a UMI count matrix comprising of number of molecules for each feature (ie gene in rows) detected per cell (columns). The UMI count matrix was used to create a Seurat object with cell-level filtering criteria as follows: (i) number of UMI > 500, (ii) number of genes ranging from 200 to 7,000, (iii) log_10_GenesPerUMI > 0.9, (iv) percentage mitochondrial count <5%, and (v) percentage chloroplast content <0.5%. Gene-level filtering was set to keep only the genes detected in at least 3 cells. The droplets potentially containing more than 1 cell were removed with doubletFinder v2.0.3 (McGinnis et al. 2019) utilizing the first 20 principal components. The optimal neighborhood size (pK) was calculated using paramSweep_v3. Considering an 8.0% doublet formation rate, expected real doublets (nExp) were estimated by integrating artificial doublets at a defined proportion (pN). In brief, the values of nExp, pN, and pK were set to 0.25, 0.26, and 4046 for the control dataset, and 0.25, 0.3, and 3,879 for the nitrate dataset, respectively. Following this, only “singlets” were kept for further analysis. The cell cycle scores were calculated by the CellCycleScoring() and PercentageFeatureSet() functions in Seurat, based on the *P. tremula* homologs for the genes defined in *Arabidopsis* (Gutierrez 2009; Zhang et al. 2021). Normalization by the regularized negative binomial regression method was done by the SCTransform() function in Seurat with 3,000 highly variable features and regressing out the variation due to cell cycle. Genes induced by protoplasting were identified as the closest homologs of those previously defined in *Arabidopsis* (Yadav et al. 2009; Chen et al. 2021) (Supplemental Data Set S3j).

Cluster-specific markers were determined using the Wilcoxon test in the FindAllMarkers() function in Seurat with genes expressed in at least 25% of all cells in a cluster and LFC > 0.25. Cluster-wise GO enrichment was achieved using topGO() function in R. The *P*-values obtained from the GO test were adjusted by Benjamini–Hochberg (BH) method for multiple testing correction (*P*_adj_ < 0.01). Genes expressed in at least 3 cells per sample were used as the background gene set for GO enrichment analysis.

Developmental trajectories for fibers and vessels were created using Monocle3 v1.4.26. A subset of clusters was extracted, and unsupervised clustering was repeated at a resolution of 0.001. Graphs were constructed using learn_graph() followed by inference of pseudotime trajectory using order_cells() and plotted using plot_cells() function. The dynamic expression of genes along pseudotime were visualized by ggplot2 v3.5.2.

#### Differential gene expression

The CCA method was used to integrate the scRNA-seq datasets from the control and nitrate treatment, which is highly recommended as it helps to match the shared cell types across conditions, boosts the statistical power, and facilitates the comparative analysis (Butler et al. 2018). After scaling the integrated data by the VST method based on the top 2,000 highly variable genes and linear dimensionality reduction by PCA, the first 50 PCs were used in a graph-based approach to identify cell clusters that were visualized by Uniform Manifold Approximation and Projection (UMAP). Only 18 clusters, showing correlation to the previous spatial or cellular *Populus* transcriptomes or marker genes, were kept for differential gene expression analysis. The gene expression variability in different cell types in response to nitrate treatment was inferred using Wilcoxon test in the FindMarkers() function of Seurat with LFC > 1.0 and FDR < 0.01 in at least 10% of all the cells in a cluster. Significantly enriched GO terms under nitrate treatment were examined as described earlier.

An interactive web application to explore the cellular transcriptome is available at https://aspcell-nitrate.serve.scilifelab.se/app/aspcell-nitrate.

### Cultivation of the hybrid aspen *CRF4* overexpression lines

Hybrid aspen wild-type cuttings (clone T89) and *LMX5pro:CRF4* (CRF4-OE) lines 2 and 9, described earlier (Vahala et al. 2013; Seyfferth et al. 2021), were cultivated for 10 wk in greenhouse conditions. Rooted cuttings, generated in vitro, were transplanted into 3-liter pots with a soil mixture of peat:perlite (10:1), mixed with 2 g lime per liter. The pot substrate (Solmull, Hasselfors Garden, Sweden) was treated with 40 mg nitrogen per liter, using RIKA-S (SW HORTO AB, Sweden; 7-1-5 N:P:K) based fertilizer. Trees were subjected to 18:6 h day/night cycles, with a daily temperature of ∼22 °C and 50 to 60% relative humidity. Ambient light was supplemented with Fionia FL300 Sunlight LED lamps (Senmatic A/S, Denmark) with a maximal irradiance of 150 to 250 µmol m^−2^ s^−1^. Trees were fertilized at 1- or 2-d intervals, with RIKA-S-based nutrient solution and NH_4_NO_3_ as a nitrogen source, the total nitrogen amount, added during the whole experiment, reaching either 0.49 g or 1.45 g.

### Cultivation and analyses of the hybrid aspen CRISPR-Cas9 lines targeting *CRF3* and *CRF4*

The hybrid aspen wild-type cuttings (clone T89) and the CRISPR-Cas9 lines 35 and 43, targeting both *CRF4* and *CRF3,* were cultivated in peat-based substrate for 11 wk in a walk-in chamber (18:6 h day/night cycles with a daily temperature of ∼22 °C and 70% relative humidity). Trees were grown for 7 wk under optimal nitrogen conditions followed by 4 wk of nitrogen starvation prior to applications with nitrate. The optimal nitrogen conditions, as defined in Renström et al. (2024), consisted of fertilization with 50 mL RIKA-S (SW HORTO AB) thrice a week, starting with a concentration of 58 mM NH_4_NO_3_ and ending with a concentration of 323 mM NH_4_NO_3_, resulting in a total of ∼1 g nitrogen supplied to each tree by the end of 7th week. After 4 additional weeks of nitrogen depletion, 5 mM KNO_3_ or 5 mM KCl (as control) was continuously injected into the xylem sap using one 60-mL syringe connected to 1.2 mm needles. The total volumes that were taken up by the trees varied between 50 to 60 mL, corresponding to a total amount of 3.14 to 4.2 mg nitrogen. After 72 h of treatments, stem pieces were harvested at the height of 5 to 40 cm above the injection point for protoplast isolation and at the height of 40 to 50 cm for RNA extraction. Nitrate in the xylem sap was measured using a protocol by Miranda et al. (2001). For RNA, the surface of the woody tissue was scraped from 2 to 3 replicate trees per genotype as described above and homogenized to a fine powder. RNA was extracted using Spectrum Plant Total RNA Kit (Sigma-Aldrich), sequenced on Illumina NovaSeq X Plus with paired-end setup (300 bp), and pre-processed for analysis as described above.

### Protoplast size estimation

Feret diameters (in µm) were measured using ImageJ. For the time-series experiment, cells were pooled from 5 trees for each treatment (control as well as 24 and 48 h after nitrate treatment). For the CRISPR-Cas9 lines, cells were analyzed from 2 to 3 independent trees for each treatment (control and nitrate). Protoplasts smaller than 10 µm in diameter were excluded from the analysis. Differences in protoplast size between the control and nitrate treatment for each genotype or time point were analyzed using a linear mixed-effects (LMM) model, with treatment and genotype or time point included as fixed effects and sample identity as a random effect to account for biological replicate structure. Complementing the LMM model, the magnitude of the nitrate-induced effect on cell expansion for each genotype or time point was estimated using rank-biserial correlation (*r*) calculated from Wilcoxon–Mann–Whitney comparisons. This correlation provides a nonparametric estimate of treatment-associated shifts in cell-size distribution and is independent of sample size. To further evaluate nitrate-induced effects on the vessel cell population, the same statistical analyses were performed on the largest 10% of cells within each genotype or time point per treatment group, defined based on the 90th percentile of the Feret diameter distributions.

### Wood anatomy

Samples were collected 20 to 25 cm above the base of the stem. Transverse sections were cut right above the node at a thickness of 30 µm with Vibratome VT100 (Leica Microsystems, Wetzlar, Germany). For vessel measurements, sections were stained with 0.05% Toluidine blue O and imaged in bright field mode with DMi8 microscope (Leica). For fibers, fluorescence images were acquired (Ex: 490 nm, DC: 510 nm EM: LP515 nm) with Leica DFC9000GT monochromatic camera and LasX software. Images were processed in ImageJ by the “analyze particle” function counting numbers of vessels, fibers, and their lumen area.

### OPTIR microspectroscopy

Wild-type hybrid aspen trees (clone T89) were grown for 7 wk under optimal nitrogen fertilization corresponding to a total dose of ∼1 g nitrogen and starved for 4 wk, after which 5 mM KNO_3_ or 5 mM KCl (as control) was applied continuously with syringes, as described above for the CRISPR-Cas9 lines. Stem samples were collected 3 cm above the injection point 72 h after nitrate application, fixed overnight in a mixture of 4% paraformaldehyde (PFA) and 0.25% glutaraldehyde in 1× phosphate-buffered saline (PBS), and stored in 1% PFA. Fifteen-micrometer transverse sections were taken with a vibratome and mounted on rectangular CaF2 slides (Crystram Ltd, UK), identical in size and shape to normal microscopy slides but transparent in infrared light. Spectra were recorded from the first secondary xylem element exhibiting signs of cell expansion in each file of the secondary xylem cells using a mIRage-LS spectrometer (Photothermal Corp, USA), equipped with a 3-chip quantum cascade laser (covering the 800 to 1,800 cm^−1^ region) as an IR pulse source and a 532-nm green laser as the probe. The IR power was kept at 20%, and the probe power was 3.4%, using the standard detector with 10× gain. A 40× Cassegrain objective was used in co-propagation mode, using the factory set “High SNR” recipe, which yields a spectral resolution of 4 cm^−1^. Multiple sections from multiple (at least 3) biological replicates were recorded, with at least 5 spectra/replicate. Spectra were exported as spc files, compiled into a single Excel file and thereafter processed with free, open-source, Matlab-based graphical user interface available via the Vibrational Spectroscopy Core Facility at Umeå University (https://www.umu.se/en/research/infrastructure/visp/downloads/). The spectral range was kept (800 to 1,800cm^−1^), and no baseline correction was needed due to the superior performance of the OPTIR recordings. However, due to the mild powers applied (to avoid sample burning), Savitzky-Golay smoothing (first-order polynomial, with a frame of 5) was applied. Spectra were total area normalized to avoid improper focus, varying sample thickness or different cell wall thickness to influence the results. The processed spectra were peak fitted between 1,480 and 1,800 cm^−1^, focusing on the -C = O bands at 1,740 and 1,600 cm^−1^, using built-in MATLAB functions (and the Optimization Toolbox). The peak model used was pseudo-Voigt V(*x*) = *A* * [eta * L(*x*) + (1 − eta) * G(*x*)], with L and G being the Lorentzian and Gaussian contributions, respectively, and eta being the mixing ratio (fitted). The peak fitting (deconvolution) results were stored for each band, including the band position (to allow the determination of band shifts), band widths at half-height (to investigate broadenings) and band integrals (ie the total area of the fitted peak, to investigate intensity variations as a measure for relative concentration changes). The areas of the 1,740 and 1,600 cm^−1^ bands were used to estimate the proportion of esterified and nonesterified pectins, respectively, in the total cell wall matrix. Significant differences were tested by Cohen's *d* values and Student's *t*-test. Box plots, including jittered points for the individual values, were used for visualizations. The *y*-axis of the box plots represents intensities (integrals in arbitrary, uniformly scaled units, as a result of the total area normalization).

## Supplementary Material

koag281_Supplementary_Data

## Data Availability

Time-series RNA-seq data are available under the ENA project ID# PRJEB78718 and the continuous 72-h nitrate treatment under the NCBI project ID# PRJNA1494053. Single-cell RNA-seq data and clustered Seurat objects for all the samples including replicates are accessible under the NCBI GEO series ID# GSE304804. Code for RNA-seq data analysis and gene co-expression network can be found at https://github.com/UPSCb/ShortTermNitrateTimeSeries-SNRIV and for scRNA-seq data analysis at https://github.com/shruti281989/SNRIII. An interactive web application to explore the cellular transcriptome is available at https://aspcell-nitrate.serve.scilifelab.se/app/aspcell-nitrate.
