# Supplementary material for: Nitrate transiently rewires transcription in developing xylem of Populus wood to promote cell expansion via CRF4
Source: Plant Cell. 2026 Sep 18;38(10):koag281. doi: 10.1093/plcell/koag281 (PMC13678210; doi:10.1093/plcell/koag281)
Supplement: Supplementary file 1 [file koag281_supplementary_data.zip › Figure S1.pdf]

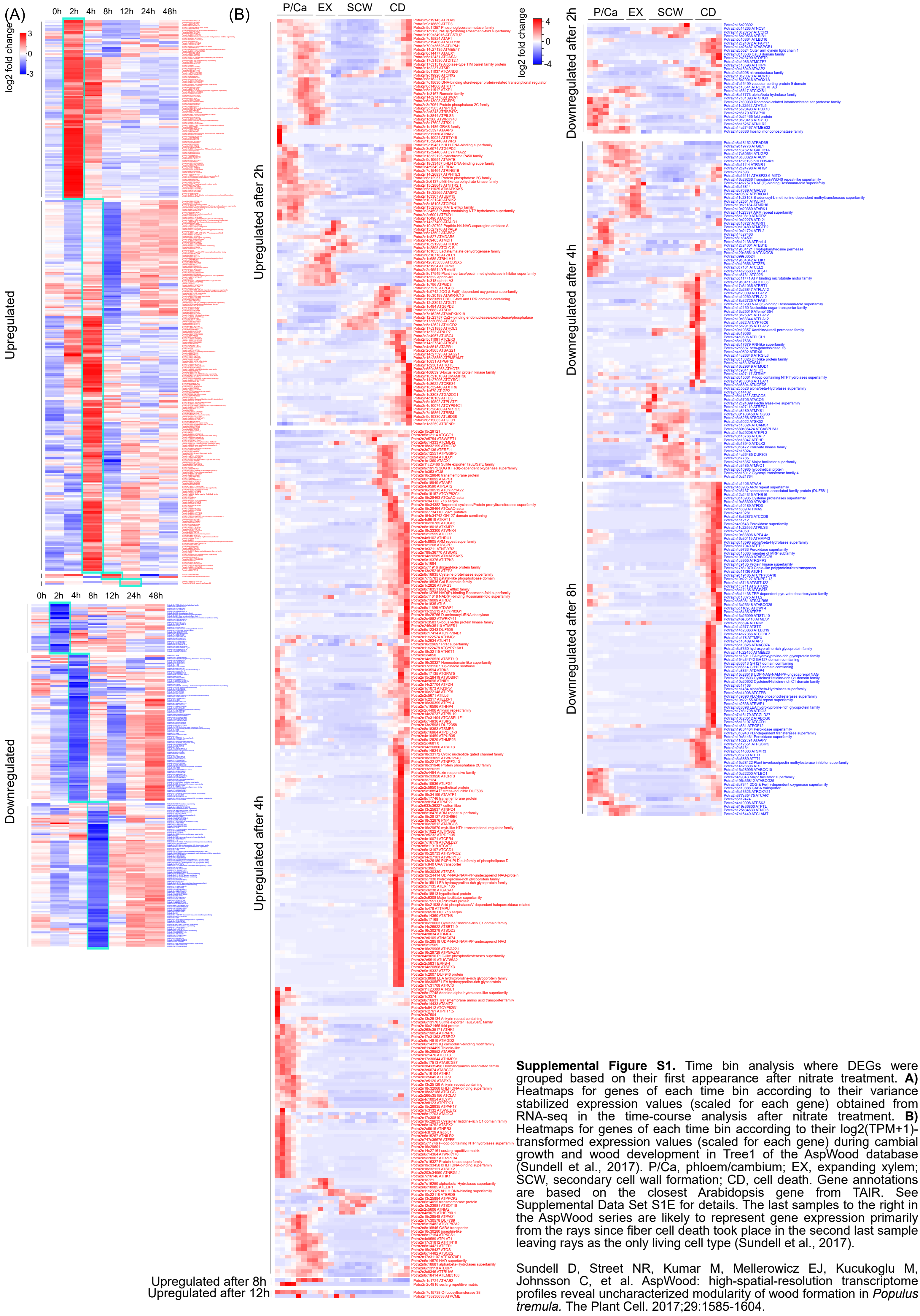

Supplemental Figure S1. Time bin analysis where DEGs were grouped based on their first appearance after nitrate treatment. **A)** Heatmaps for genes of each time bin according to their variance stabilized expression values (scaled for each gene) obtained from RNA-seq in the time-course analysis after nitrate treatment. **B)** Heatmaps for genes of each time bin according to their log2(TPM+1)-transformed expression values (scaled for each gene) during cambial growth and wood development in Tree1 of the AspWood database (Sundell et al., 2017). P/Ca, phloem/cambium; EX, expanding xylem; SCW, secondary cell wall formation; CD, cell death. Gene annotations are based on the closest Arabidopsis gene from TAIR. See Supplemental Data Set S1E for details. The last samples to the right in the AspWood series are likely to represent gene expression primarily from the rays since fiber cell death took place in the second last sample leaving rays as the only living cell type (Sundell et al., 2017).

Sundell D, Street NR, Kumar M, Mellerowicz EJ, Kucukoglu M, Johnsson C, et al. AspWood: high-spatial-resolution transcriptome profiles reveal uncharacterized modularity of wood formation in *Populus tremula*. The Plant Cell. 2017;29:1585-1604.
