# Supplementary material for: Nitrate transiently rewires transcription in developing xylem of Populus wood to promote cell expansion via CRF4
Source: Plant Cell. 2026 Sep 18;38(10):koag281. doi: 10.1093/plcell/koag281 (PMC13678210; doi:10.1093/plcell/koag281)
Supplement: Supplementary file 1 [file koag281_supplementary_data.zip › Figure S10.pdf]

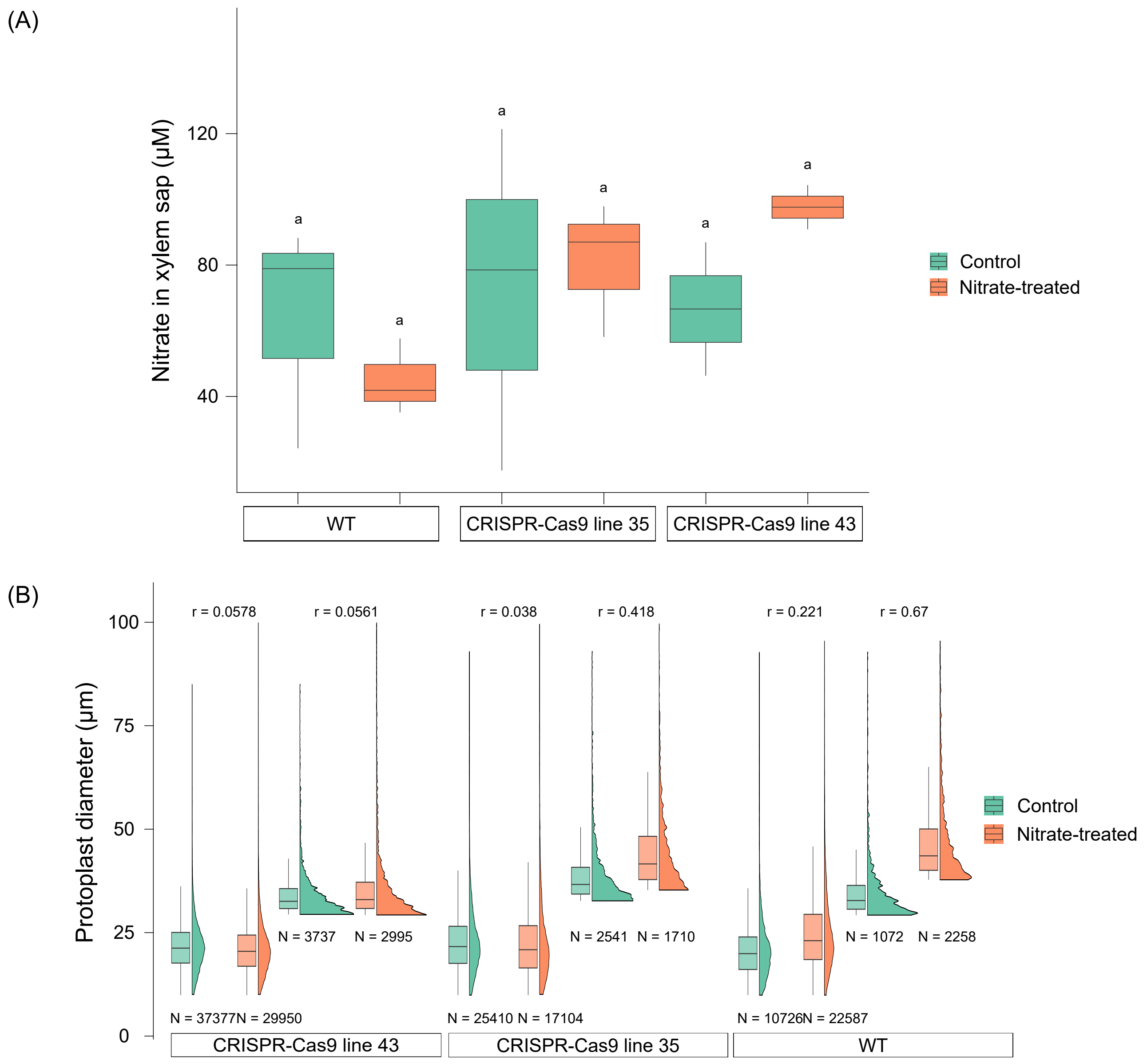

Supplemental Figure S10. Further details on the CRISPR-Cas 9 lines 35 and 43 targeting *CRF4* and *CRF3* in hybrid aspen trees. **A)** Concentration of nitrate in xylem sap in wild type and the CRISPR-Cas9 lines 35 and 43 in response to nitrate treatment in Figure 7B. Statistical significance, comparing treatment for each genotype, was tested with Two Sample t-test. Treatment of 5 mM KCl was used as a control. Means not sharing any letter are significantly different at 5% level of significance. **B)** Cell sizes in developing xylem of stem tissues in wild type and the CRISPR-Cas9 lines 35 and 43 in response to nitrate treatment. Trees were grown in peat-based soil with a total of ~0.606 g of nitrogen for seven weeks and depleted for nitrogen during the four weeks before nitrate treatment. 5 mM KNO3 or 5 mM KCl (as control) was continuously injected into the base of the stem. Protoplasts were isolated from developing stem tissues 72 h after treatment and measured for the diameter. N = number of protoplasts from the developing xylem taken for size measurements from 2-3 individual trees per treatment for each line. Statistics were performed for all cells as well as the largest 10% of the protoplast population (shown alongside all cells) within each genotype and treatment. The magnitude of nitrate-induced effects on cell expansion as an effect size was measured using rank-biserial correlation (r), calculated from Wilcoxon–Mann–Whitney comparisons.
