# Supplementary material for: Nitrate transiently rewires transcription in developing xylem of Populus wood to promote cell expansion via CRF4
Source: Plant Cell. 2026 Sep 18;38(10):koag281. doi: 10.1093/plcell/koag281 (PMC13678210; doi:10.1093/plcell/koag281)
Supplement: Supplementary file 1 [file koag281_supplementary_data.zip › Figure S2.pdf]

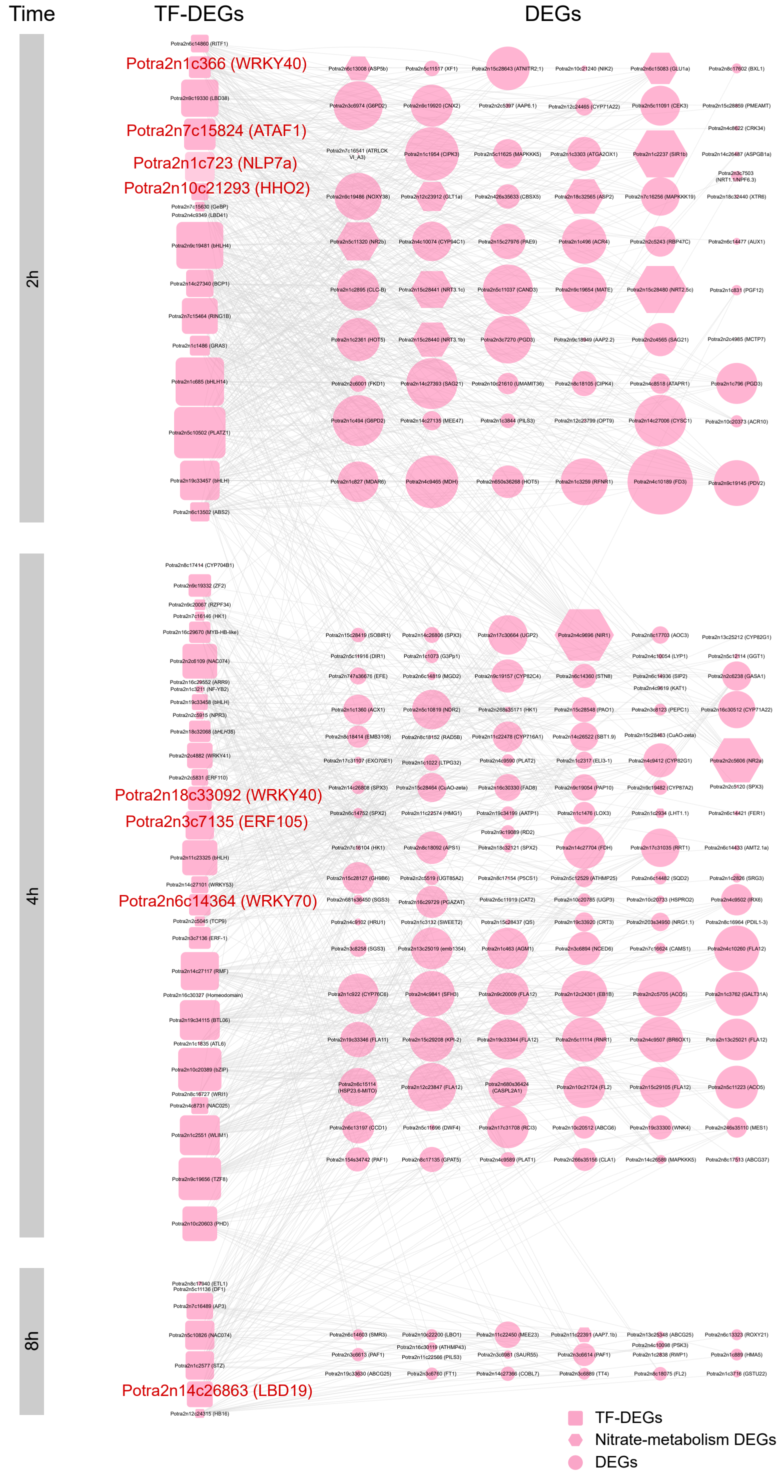

Supplemental Figure S2. A transcription factor co-expression network in the developing xylem tissues of hybrid aspen. The network is based on differentially expressed transcription factors (denoted as TF-DEGs) and other DEGs that were first-degree neighbors of the TF-DEGs (denoted as DEGs) in the time-course analysis of gene expression after nitrate treatment. Only edges between TF-DEGs and DEGs are shown. Genes involved in nitrate metabolism are indicated. The size of the node is directly proportional to its degree i.e., the number of directly connected genes. Seven TF-DEGs with a significant nitrogen-specificity index ($P<0.05$) are marked in red. See Supplemental Data Set S1H for details.
