# Supplementary material for: Nitrate transiently rewires transcription in developing xylem of Populus wood to promote cell expansion via CRF4
Source: Plant Cell. 2026 Sep 18;38(10):koag281. doi: 10.1093/plcell/koag281 (PMC13678210; doi:10.1093/plcell/koag281)
Supplement: Supplementary file 1 [file koag281_supplementary_data.zip › Figure S3 smaller.pdf]

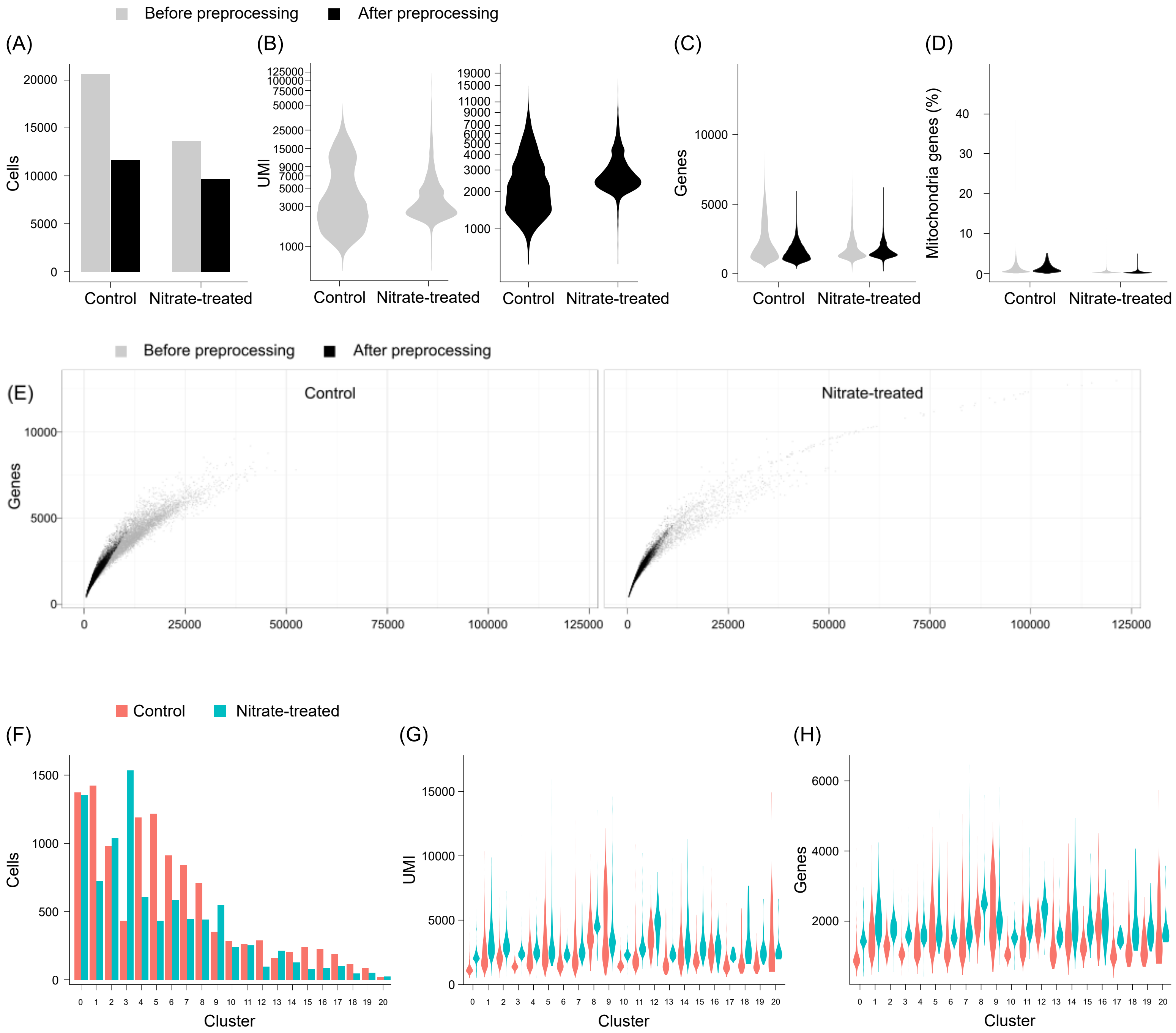

Supplemental Figure S3. Assessment of quality parameters for scRNA-seq data. **A-E)** scRNA-seq data for control and nitrate-treated sample was processed to remove low quality cells based on number of UMI, genes, mitochondrial gene content and doublets. Plots show total number of cells (A), UMI (B), genes (C), percentage of mitochondrial reads (D), and number of UMI detected vs genes (E) per sample before and after preprocessing. **F-H)** Stats for the final integrated data from the two samples include, number of cells (F), UMI counts (G), and gene counts (H) per cluster per sample.
