# Supplementary material for: Nitrate transiently rewires transcription in developing xylem of Populus wood to promote cell expansion via CRF4
Source: Plant Cell. 2026 Sep 18;38(10):koag281. doi: 10.1093/plcell/koag281 (PMC13678210; doi:10.1093/plcell/koag281)
Supplement: Supplementary file 1 [file koag281_supplementary_data.zip › Figure S4.pdf]

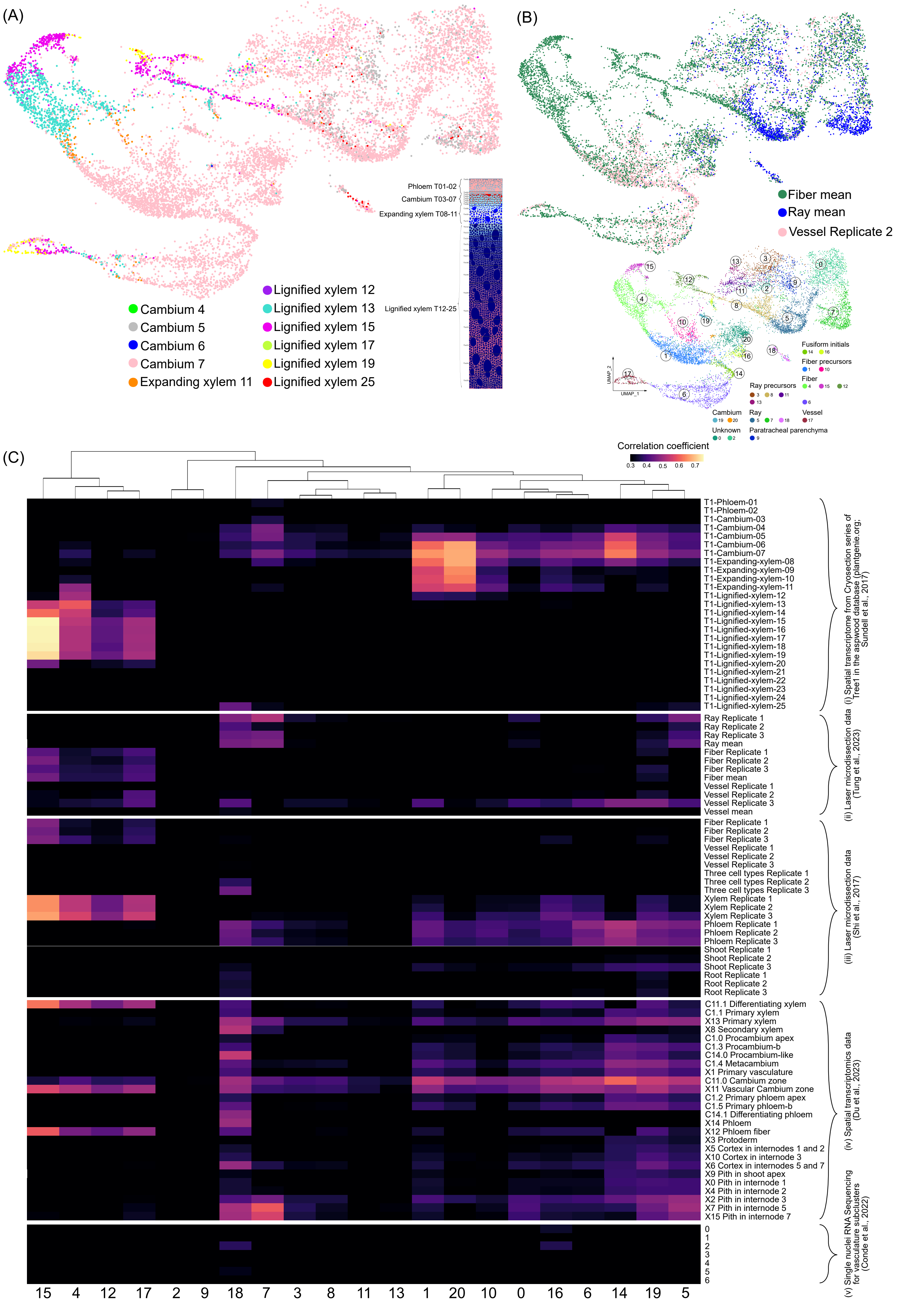

(A)
Phloem T01-02
Cambium T03-07
Expanding xylem T08-11
Lignified xylem T12-25
Cambium 4
Cambium 5
Cambium 6
Cambium 7
Expanding xylem 11
Lignified xylem 12
Lignified xylem 13
Lignified xylem 15
Lignified xylem 17
Lignified xylem 19
Lignified xylem 25
(B)
Fiber mean
Ray mean
Vessel Replicate 2
UMAP_2
UMAP_1
Fusiform initials
Fiber precursors
Fiber
Ray precursors
Cambium
Ray
Vessel
Unknown
Paratracheal parenchyma
(C)
Correlation coefficient
0.3 0.4 0.5 0.6 0.7
T1-Phloem-01
T1-Phloem-02
T1-Cambium-03
T1-Cambium-04
T1-Cambium-05
T1-Cambium-06
T1-Cambium-07
T1-Expanding-xylem-08
T1-Expanding-xylem-09
T1-Expanding-xylem-10
T1-Expanding-xylem-11
T1-Lignified-xylem-12
T1-Lignified-xylem-13
T1-Lignified-xylem-14
T1-Lignified-xylem-15
T1-Lignified-xylem-16
T1-Lignified-xylem-17
T1-Lignified-xylem-18
T1-Lignified-xylem-19
T1-Lignified-xylem-20
T1-Lignified-xylem-21
T1-Lignified-xylem-22
T1-Lignified-xylem-23
T1-Lignified-xylem-24
T1-Lignified-xylem-25
(i) Spatial transcriptome from Cryosection series of Tree1 in the aspwood database (plantgenie.org; Sundell et al., 2017)
Ray Replicate 1
Ray Replicate 2
Ray Replicate 3
Ray mean
Fiber Replicate 1
Fiber Replicate 2
Fiber Replicate 3
Fiber mean
Vessel Replicate 1
Vessel Replicate 2
Vessel Replicate 3
Vessel mean
(ii) Laser microdissection data (Tung et al., 2023)
Fiber Replicate 1
Fiber Replicate 2
Fiber Replicate 3
Vessel Replicate 1
Vessel Replicate 2
Vessel Replicate 3
Three cell types Replicate 1
Three cell types Replicate 2
Three cell types Replicate 3
Xylem Replicate 1
Xylem Replicate 2
Xylem Replicate 3
Phloem Replicate 1
Phloem Replicate 2
Phloem Replicate 3
Shoot Replicate 1
Shoot Replicate 2
Shoot Replicate 3
Root Replicate 1
Root Replicate 2
Root Replicate 3
(iii) Laser microdissection data (Shi et al., 2017)
C11.1 Differentiating xylem
C1.1 Primary xylem
X13 Primary xylem
X8 Secondary xylem
C1.0 Procambium apex
C1.3 Procambium-b
C14.0 Procambium-like
C1.4 Metacambium
X1 Primary vasculature
C11.0 Cambium zone
X11 Vascular Cambium zone
C1.2 Primary phloem apex
C1.5 Primary phloem-b
C14.1 Differentiating phloem
X14 Phloem
X12 Phloem fiber
X3 Protoderm
X5 Cortex in internodes 1 and 2
X10 Cortex in internode 3
X6 Cortex in internodes 5 and 7
X9 Pith in shoot apex
X0 Pith in internode 1
X4 Pith in internode 2
X2 Pith in internode 3
X7 Pith in internode 5
X15 Pith in internode 7
(iv) Spatial transcriptomics data (Du et al., 2023)
0
1
2
3
4
5
6
(v) Single nuclei RNA Sequencing for vasculature subclusters (Conde et al., 2022)
15 4 12 17 2 9 18 7 3 8 11 13 1 20 10 0 16 6 14 19 5

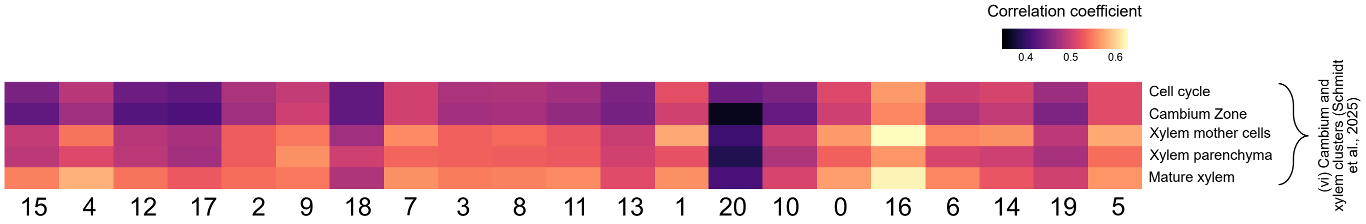

Supplemental Figure S4. Correlation of clusters identified from our scRNA-seq experiment with reported spatial and cellular transcriptomes in *Populus* species. **A)** UMAP showing the individual cells from our scRNA-seq data with the highest correlation to the cryosections from Tree1 in the Aspwood database (Sundell et al., 2017). **B)** UMAP showing the individual cells from our scRNA-seq data with the highest correlation to the transcriptome profiles of laser capture microdissected (LCM) xylem rays, fibers, and vessels from *P. trichocarpa*, reported in Tung et al. (2023). Note that for the vessels, we show only replicate 2 that most likely is free from other cell types. The color shows the correlation to the highest correlation value. UMAP along with cluster annotation for our scRNA-seq data is shown reference at the bottom. **C)** Correlation heatmap of our scRNA-seq clusters (below) to various cellular and spatial profiles available in the public domain. (i) Correlation to RNA-seq data from Aspwood cryosections from Tree 1 where sections 1 and 2 consist of phloem, 3-7 vascular cambium, 8-11 expanding xylem and the rest lignified xylem. Vessel cell death is estimated to occur in section 18 and fiber cell death in section 24. Section 25 comprises exclusively of living rays. (ii) Correlation to RNA-seq data of three biological replicates of each of the xylem cell type (fiber, vessel and ray) captured by LCM and their respective means from Tung et al. (2023). (iii) Correlation to RNA-seq data of various xylem cells and tissues captured by LCM in Shi et al. (2017). (iv) Correlation to clusters obtained from spatial transcriptome profiles from shoot apex till the 7th internode of wood by Du et al. (2023). (v) Correlation to clusters obtained from single nuclei RNA-seq from vasculature of the shoot apex by Conde et al. (2022). (vi) Correlation to clusters obtained from single nuclei RNA-seq from cambium and xylem clusters by Schmidt et al. (2025). Gene expression data utilized in the analysis are $\log_2$(TPM+1)-transformed expression values.

1. Conde D, Triozzi PM, Pereira WJ, Schmidt HW, Balmant KM, Knaack SA, et al. Single-nuclei transcriptome analysis of the shoot apex vascular system differentiation in Populus. Development. 2021,149:dev200632.
2. Du J, Wang Y, Chen W, Xu M, Zhou R, Shou H, et al. High-resolution anatomical and spatial transcriptome analyses reveal two types of meristematic cell pools within the secondary vascular tissue of poplar stem. Mol Plant. 2023,16:809-828.
3. Shi R, Wang JP, Lin Y-C, Li Q, Sun Y-H, Chen H, et al. Tissue and cell-type co-expression networks of transcription factors and wood component genes in Populus trichocarpa. Planta. 2017;245:927-938.
4. Sundell D, Street NR, Kumar M, Mellerowicz EJ, Kucukoglu M, Johnsson C, et al. AspWood: high-spatial-resolution transcriptome profiles reveal uncharacterized modularity of wood formation in Populus tremula. The Plant Cell. 2017;29:1585-1604.
5. Tung C-C, Kuo S-C, Yang C-L, Yu J-H, Huang C-E, Liou P-C, et al. Single-cell transcriptomics unveils xylem cell development and evolution. Genome Biol. 2013,24:3.
6. Schmidt HW, Conde D, Pereira WJ, Triozzi PM, Balmant KM, Dervinis C, Kirst M. Deep tissue profiling of Populus stem at single nucleus level reveals uncharacterized cell types and cell-specific gene regulatory networks. Genome Biology. 2025;26:258.
