# Supplementary material for: Nitrate transiently rewires transcription in developing xylem of Populus wood to promote cell expansion via CRF4
Source: Plant Cell. 2026 Sep 18;38(10):koag281. doi: 10.1093/plcell/koag281 (PMC13678210; doi:10.1093/plcell/koag281)
Supplement: Supplementary file 1 [file koag281_supplementary_data.zip › Figure S5.pdf]

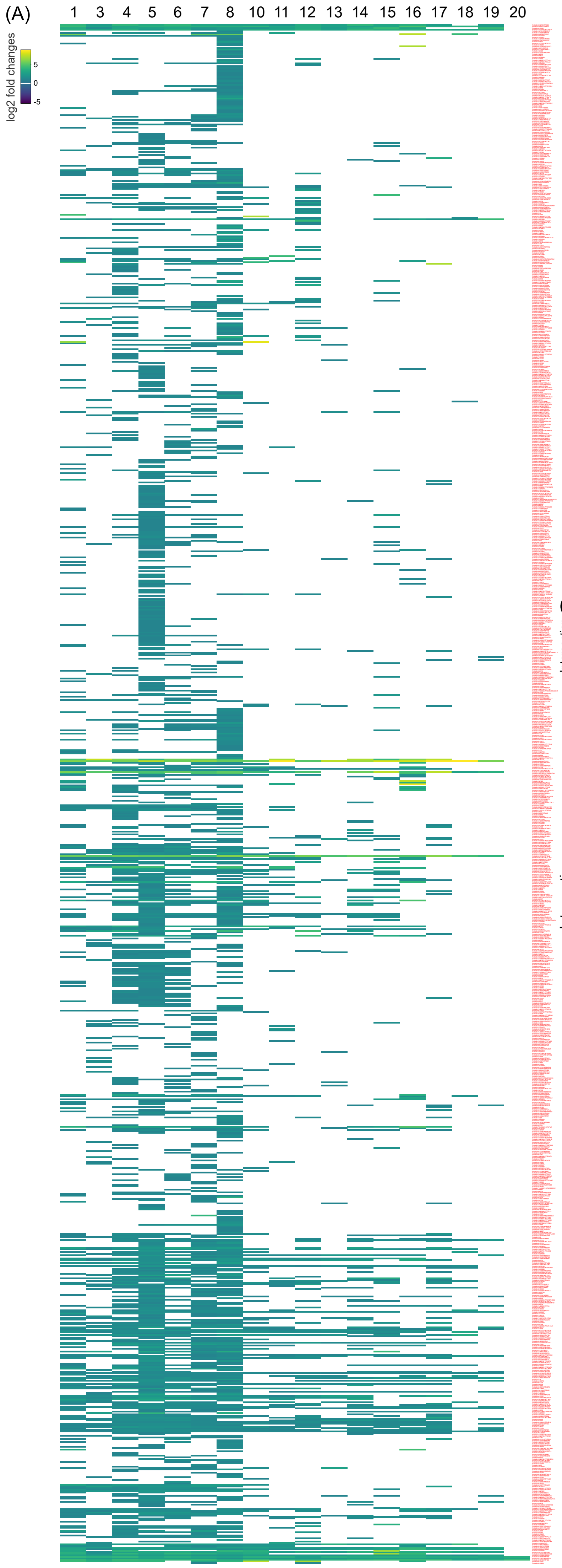

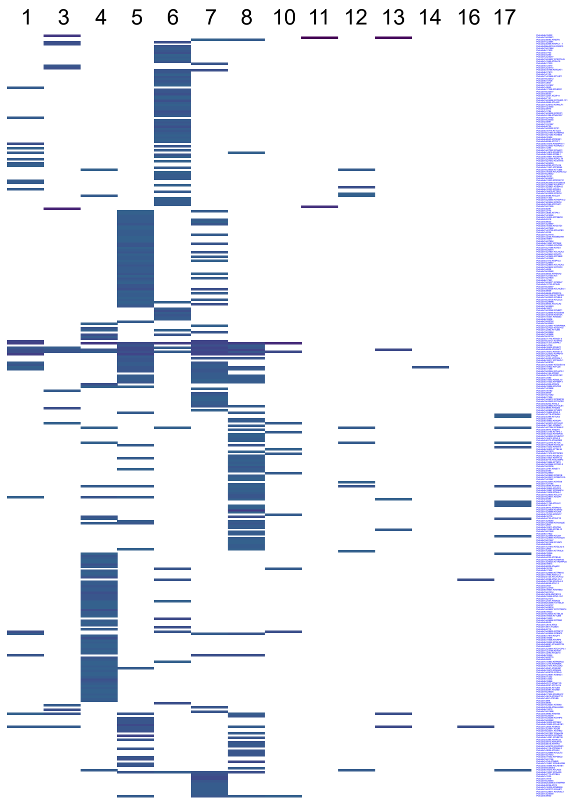

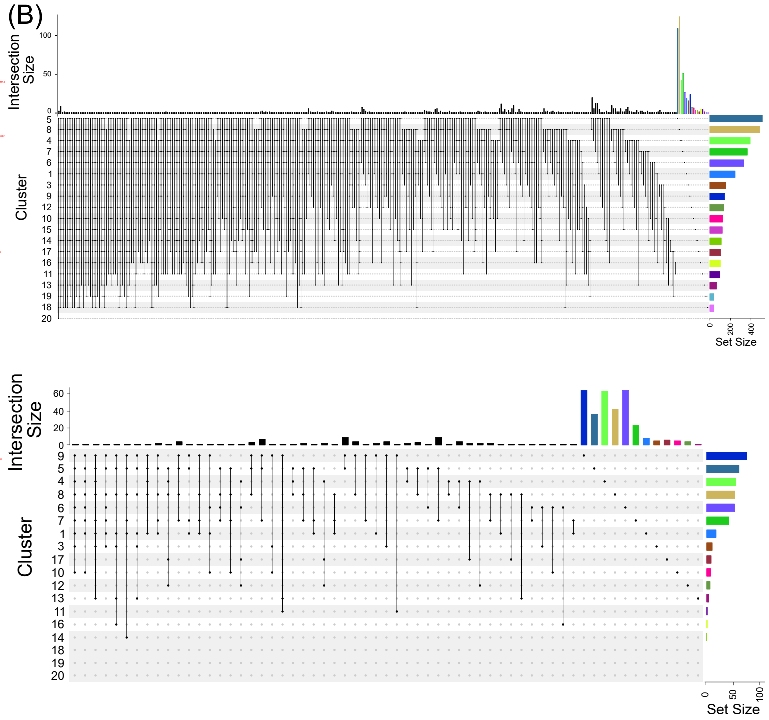

Supplemental Figure S5 Gene expression in response to nitrate in xylem cells. **A)** Heatmap for cluster-wise expression profile showing log_2 fold change (LFC) of differentially expressed genes (DEGs) for the comparison between nitrate treatment versus control. DEGs fitting the significance criteria (LFC >1 and FDR <0.01) in a specific cluster are shown with up and downregulated genes in red and blue, respectively. Gene annotations are based on the closest *Arabidopsis* homologs. **B)** UpSet plot showing the unique and shared number of upregulated (upper panel) and downregulated (lower panel) genes identified in each cluster for nitrate versus control treatment. See Supplemental Data Set S3E for more details.
