# Supplementary material for: Nitrate transiently rewires transcription in developing xylem of Populus wood to promote cell expansion via CRF4
Source: Plant Cell. 2026 Sep 18;38(10):koag281. doi: 10.1093/plcell/koag281 (PMC13678210; doi:10.1093/plcell/koag281)
Supplement: Supplementary file 1 [file koag281_supplementary_data.zip › Figure S6.pdf]

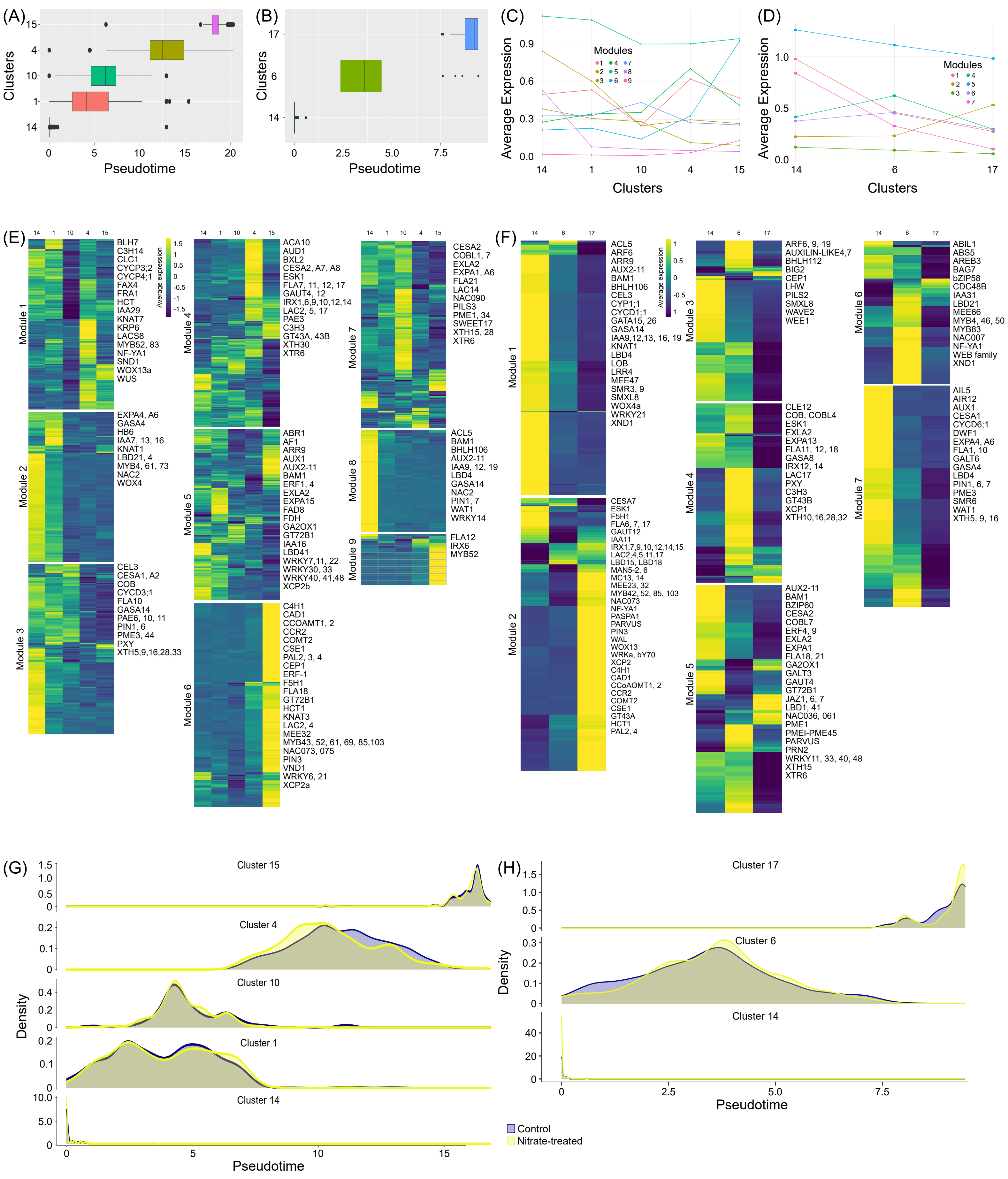

Supplemental Figure S6. Pseudotime developmental trajectory for xylem fiber and vessels. **A-B)** Pseudotime analysis showing differentiation from fusiform initials cluster 14 to fiber clusters 1, 10, 4 and 15 (A) and vessel clusters 6 and 17 (B). **C-D)** Gene modules that co-vary across pseudotime during fiber (C) and vessel (D) cell differentiation. **E-F)** Heatmap of pseudotime-dependent genes in each module during fiber (E) and vessel (F) differentiation. Example of genes in each module are shown. See Supplemental Data Set S3G for full set of genes in each module. **G-H)** Density plot showing fiber (G) and vessel (H) developmental trajectories for control and nitrate-treated samples over pseudotime.
