# Supplementary material for: Nitrate transiently rewires transcription in developing xylem of Populus wood to promote cell expansion via CRF4
Source: Plant Cell. 2026 Sep 18;38(10):koag281. doi: 10.1093/plcell/koag281 (PMC13678210; doi:10.1093/plcell/koag281)
Supplement: Supplementary file 1 [file koag281_supplementary_data.zip › Figure S7.pdf]

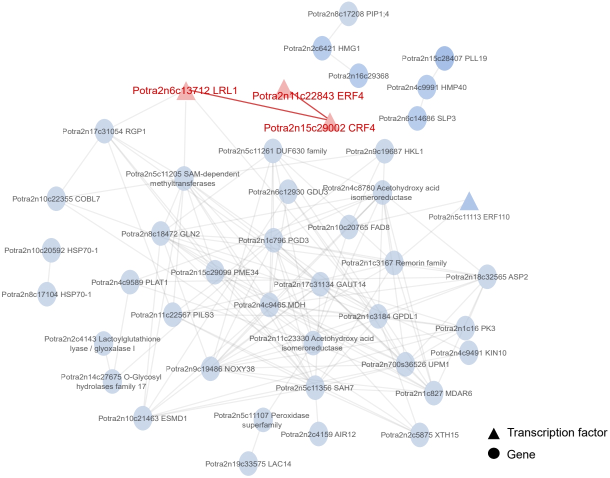

Supplemental Figure S7. A sub-network of vessel-cell expansion. The sub-network was extracted from the whole-transcriptome co-expression network, constructed based on the data in the RNAseq of the time series. The sub-network contains TF-DEGs and their first-degree DEG neighbors that were specifically expressed in the phase of xylem expansion in the AspWood database and present in the scRNAseq vessel trajectory. Genes co-expressed with *CRF4* are highlighted in red.
