# Supplementary material for: Nitrate transiently rewires transcription in developing xylem of Populus wood to promote cell expansion via CRF4
Source: Plant Cell. 2026 Sep 18;38(10):koag281. doi: 10.1093/plcell/koag281 (PMC13678210; doi:10.1093/plcell/koag281)
Supplement: Supplementary file 1 [file koag281_supplementary_data.zip › Figure S8R.pdf]

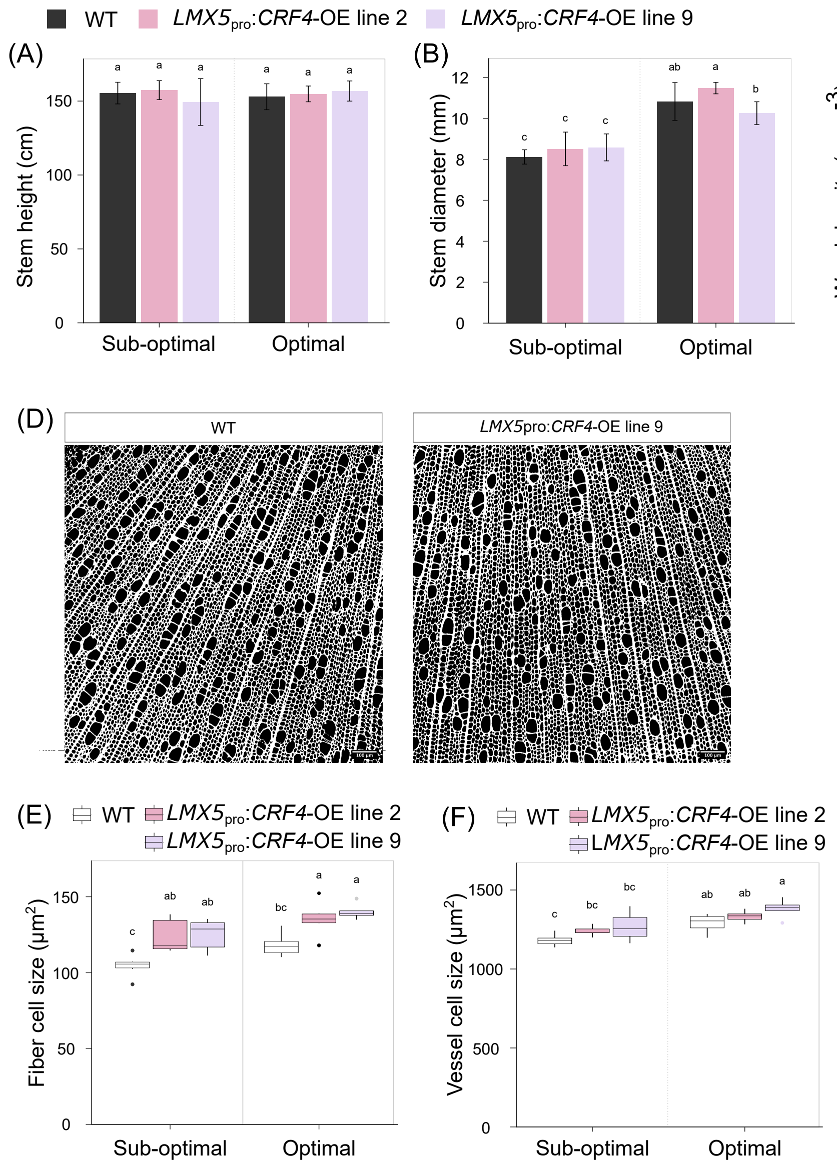

Supplemental Figure S8. *CRF4* overexpression from a wood-specific *LMX5* promoter mimics the effect of nitrate on xylem cell expansion in hybrid aspen. Two transgenic $LMX5_{pro}$:*CRF4* overexpression (*CRF4*-OE) lines and wild-type trees were grown for 10 weeks in two different fertilization regimes corresponding to a total amount of 0.49 g (sub-optimal) or 1.45 g (optimal) nitrogen. **A-C)** Overall growth of the *CRF4*-OE trees. Stem height (A), stem diameter (B) and wood density. N=5-7. **D)** Wood anatomy in the CRF4-OE trees grown for 10 weeks with optimal nitrogen. Representative images of wood in transverse sections from the base of *CRF4*-OE and wild-type stems. Bright-field images were made binary in ImageJ. Scale bar = 100 µm. **E-F)** Size of the fibers (B) and vessel elements (C) of *CRF4*-OE and WT trees in sections collected 20-25 cm from the base of the stem. N=5-7. Statistical significance, comparing WT and *CRF4*-OE trees as independent groups, is tested with two-way ANOVA and Tukey's HSD test. Means not sharing any letter are significantly different at 5% level of significance. Error bars indicate mean ± SD.
