# Supplementary material for: Nitrate transiently rewires transcription in developing xylem of Populus wood to promote cell expansion via CRF4
Source: Plant Cell. 2026 Sep 18;38(10):koag281. doi: 10.1093/plcell/koag281 (PMC13678210; doi:10.1093/plcell/koag281)
Supplement: Supplementary file 1 [file koag281_supplementary_data.zip › Figure S9.pdf]

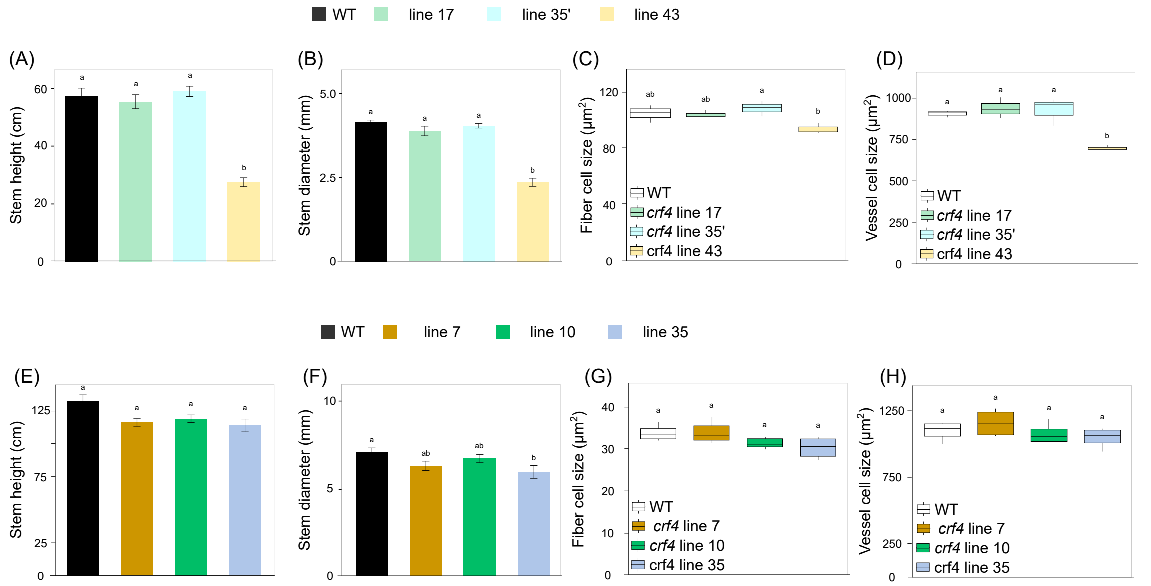

Supplemental Figure S9. Growth and wood properties of CRISPR-Cas9 lines targetting hybrid aspen *CRF4* and *CRF3*. Six CRISPR-Cas9 lines and the corresponding wild-type trees were grown for 8 weeks in greenhouse with 0.6 g nitrogen. **A to D)** Overall growth and xylem cell properties of wild type and three CRISPR-Cas9 lines from transformation #4360 (see Supplementary Table S2). **E to H**) Overall growth and xylem cell properties of wild type and three CRISPR-Cas9 lines from transformation #4424 (see Supplementary Table S2). Data is shown for stem height (A, E), stem diameter (B, F), area of xylem fibers (C, G) and area of xylem vessel elements (D, H). For fiber and vessel element areas, transverse sections were collected 5 cm from the base of the stem. N=4-6 for panels A, B, E and F. N=3-4 for panels C, D, G and H. Statistical significance, comparing WT and CRISPR-Cas9 lines as independent groups, is tested with two-way ANOVA and Tukey's HSD test. Means not sharing any letter are significantly different at 5% level of significance. Error bars indicate mean ± SD.
