# Supplementary material for: Nitrate transiently rewires transcription in developing xylem of Populus wood to promote cell expansion via CRF4
Source: Plant Cell. 2026 Sep 18;38(10):koag281. doi: 10.1093/plcell/koag281 (PMC13678210; doi:10.1093/plcell/koag281)
Supplement: Supplementary file 1 [file koag281_supplementary_data.zip › Supplementary Table S1.docx]

**Supplemental Table S1.** Primer sequences.

| **Primers** | **Name** | **Sequence (5`- 3`)** |
| --- | --- | --- |
| **Cloning oligos** | G8 Forward | ATATATGGTCTCGATTG CAGAAAGTTCCGTGGCGTT GTTTTAGAGCTAGAAATAGC |
|  | G10 Reverse | ATTATTGGTCTCTAAAC TACTAGGCGGTGTGATGAA CAATCTCTTAGTCGACTCTAC |
|  | G1 Forward | ATATATGGTCTCGATTG GTTCCGTGGCGTTCGGCAA GTTTTAGAGCTAGAAATAGC |
|  | G7 Reverse | ATTATTGGTCTCTAAAC CCCTACTAGGCGGTGTGAT CAATCTCTTAGTCGACTCTAC |
|  | G3 Forward | ATATATGGTCTCGATTG ACACCGCCTAGTAGGGAGG GTTTTAGAGCTAGAAATAGC |
|  | G6 Reverse | ATTATTGGTCTCTAAAC TGGGGTAAATGGGCTGCAG CAATCTCTTAGTCGACTCTAC |
|  | CIB promoter Forward | TAAACTCAAGCACACACGGG |
|  | CIB promoter Reverse | AAGGAAAGAAAGTAACAAGC |
|  | XCP2 promoter Forward | GAACACTTCGAGGAAGAAGG |
|  | XCP2 promoter Reverse | GTGTTGCTTCAAGTAATGTGC |
|  | F5Ha promoter Forward | TAATTGCATGAGTGCGTGGC |
|  | F5Ha promoter Reverse | GGATGCAGGTTTTTGTGTTTG |
|  |  |  |
| **Genotyping oligos** | CRF4&CRF3_FWB1 | AGTATCCTATGTCCCATAAA |
|  | CRF4& CRF3_Rv1B | CACAGATTTTGGAGATGACAG |
|  | CRF4_FW2 | CCATAAAATACACTGAACACA |
|  | CRF4_Rv1B | CACAGATTTTGGAGATGACAG |
|  | CRF3_FW3 | CCATAAAGTACACCGAGCACA |
|  | CRF3_Rv1B | CACAGATTTTGGAGATGACAG |
