# Supplementary material for: Nitrate transiently rewires transcription in developing xylem of Populus wood to promote cell expansion via CRF4
Source: Plant Cell. 2026 Sep 18;38(10):koag281. doi: 10.1093/plcell/koag281 (PMC13678210; doi:10.1093/plcell/koag281)
Supplement: Supplementary file 1 [file koag281_supplementary_data.zip › Supplementary Table S2.docx]

**Supplemental Table S2.** Genotyping for CRISPR-Cas9 lines targeting hybrid aspen *CRF3* and *CRF4*. Two different sets of guide RNAs were designed for two different transformations (4360 and 4424) The nucleotide sequences around the target sites of wild type (WT) and the CRISPR-Cas9 lines are shown for both the alleles of both *CRF4* (Potra2n15c29002; Potrx005066g03918) and *CRF3* (Potra2n12c23983, Potrx004339g03335). Target sequence of guide RNA is underlined, and PAM sequence is shown in bold. The point (.) represents the expected cut site of Cas9. Red dash (---) indicates deleted nucleotides while “+” and nucleotide shown in red represent inserted nucleotides. The length of indels is denoted and signified by minus or plus for a deletion or an insertion, respectively. The number of mutated sequences detected among the sequenced fragments for each allele is also indicated.

| **Gene** | **Line** | **Allele** | **Target sequence regions**(sgRNA, **PAM, indels**) | **Indel (bp)** | **Mutated/ Replicates** |
| --- | --- | --- | --- | --- | --- |
| **CRF4** | **WT**  **(T89)** | chr15A | TCTTGTTCTTGCT**CCT**CCT.CCCTACTAGGCGGTGTGATGAAGTTTGTTAA |  |  |
|  |  |  | GGATCTCTAATCTCTGCAGCCCATTTACC.CCA**AGG**CCTTTGCCGAACGCC |  |  |
|  |  | chr15D | TCTTGTTCTTGCT**CCT**CCT.CCCTACTAGGCGGTGTGATGAAGTTTGTTAA |  |  |
|  |  |  | GGATCTCTAATCTCTGCAGCCCATTTACC.CCA**AGG**CCTTTGCCGAACGCC |  |  |
|  | **Line 17**  **(4360#17)** | chr15A | TCTTGTTCTTGCT**CCT**CCT.C**--------------->** | -159 | 4/4 |
|  |  |  | **<---------------**.CCA**AGG**CCTTTGCCGAACGCC |  |  |
|  |  | chr15D | TC**-------------------**CTACTAGGCGGTGTGATGAAGTTTGTTAA | -19 | 4/4 |
|  |  |  | GGATCTCTAATCTCTGCAGCCCATTTACC.**-**CA**AGG**CCTTTGCCGAACGCC | -1 |  |
|  | **Line 35’**  **(4360#35)** | chr15A | TCTTGTTCTTGCT**CCT**CCT.C**--------------->** | -159 | 4/4 |
|  |  |  | **<---------------**.CCA**AGG**CCTTTGCCGAACGCC |  |  |
|  |  | chr15D | TCTTGTTCTTG**<+++++++++++++++>**.CCCTACTAGGCGGTGTGATGAA | +203 | 4/4 |
|  |  |  | GGATCTCTAATCTCTGCAGCCCATTTAC**-**.CCA**AGG**CCTTTGCCGAACGCC | -1 |  |
|  | **Line 43 (4360#43)** | chr15A | TCTTGTTCTTGCT**CCT**CCT**<++++++++>**.CCCTACTAGGCGGTGTGATGA | +128 | 4/4 |
|  |  |  | GGATCTCTAATCTCTGCAGCCCATTTACC.**--**A**AGG**CCTTTGCCGAACGCC | -2 |  |
|  |  | chr15D | TCTTGTTCTTGCT**CCT**CCT.**A**CCCTACTAGGCGGTGTGATGAAGTTTGTTA | +1 | 3/3 |
|  |  |  | GGATCTCTAATCTCTGCAGCCCATTTAC**-**.CCA**AGG**CCTTTGCCGAACGCC | -1 |  |
|  |  |  |  |  |  |
| **CRF3** | **WT**  **(T89)** | chr12A | TCTTCTTCTTGAT**CCT**CCT.CCCTACTAGGCGGTGTGATGAAATTTGTTAA |  |  |
|  |  |  | CAGGATCTCTGATCTCTGCAGCCCATTTACC.CCA**GGG**CCTTTGCCGAACG |  |  |
|  |  | chr12D | TCTTCTTCTTGAT**CCT**CCT.CCCTGCTAGGCGGTGTGATGAAATTTGTTAA |  |  |
|  |  |  | CAGGATCTCTGATCTCTGCAGCCCATTTACC.CCA**GGG**CCTTTGCCGAACG |  |  |
|  | **Line 17**  **(4360#17)** | chr12A | TCTTCTTCTTGAT**CCT**CCT.**--------------->** | -160 | 4/4 |
|  |  |  | **<---------------**.CCA**GGG**CCTTTGCCGAACG |  |  |
|  |  | chr12D | TCTTCTTCTTGAT**CCT**CCT.C**--------------->** | -159 |  |
|  |  |  | **<---------------**.CCA**GGG**CCTTTGCCGAACG |  |  |
|  | **Line 35’**  **(4360#35)** | chr12A | TCTTCTTCTTGAT**CCT**CCT.**--------------->** | -164 | 4/4 |
|  |  |  | **<---------------**CA**GGG**CCTTTGCCGAACG |  |  |
|  |  | chr12D | TCTTCTTCTTGAT**CCT**CCT.**C**CCCTGCTAGGCGGTGTGATGAAATTTGTTAA | +1 | 4/4 |
|  |  |  | CAGGATCTCTGATCTCTGCAGCCCATTTAC**-**.CCA**GGG**CCTTTGCCGAACG | -1 |  |
|  | **Line 43 (4360#43)** | chr12 | TCTTCTTCTTGAT**CCT**CCT.C**--------------->** | -159 | 4/4 |
|  |  |  | **<---------------**.CCA**GGG**CCTTTGCCGAACG |  |  |
|  |  |  | The second allele failed to be amplified, indicating a larger deletion in this genomic region. |  |  |

| **Gene** | **Line** | **Allele** | **Target sequence regions**(sgRNA, **PAM, indels**) | **Indel (bp)** | **Mutated/ Replicates** |
| --- | --- | --- | --- | --- | --- |
| **CRF4** | **WT (T89)** | chr15A | CTTGCTCCT**CCT**CCC.TACTAGGCGGTGTGATGAAGTTTGTTAAAGC |  |  |
|  |  |  | ACCCCAAGG**CCT**TTG.CCGAACGCCACGGAACTTTCTGCCATTGTTTGTT |  |  |
|  |  | chr15D | CTTGCTCCT**CCT**CCC.TACTAGGCGGTGTGATGAAGTTTGTTAAAGC |  |  |
|  |  |  | ACCCCAAGG**CCT**TTG.CCGAACGCCACGGAACTTTCTGCCATTGTTTGTT |  |  |
|  | **Line 7**  **(4424#7)** | chr15A | CTTGCTCCT**CCT**CCC.**------------>** | -169 | 4/4 |
|  |  |  | **<------------**.CCGAACGCCACGGAACTTTCTGCCATTGTTTGTT |  |  |
|  |  | chr15D | CTTGCTCCT**CCT**CCC.**-**ACTAGGCGGTGTGATGAAGTTTGTTAAAGC | -1 | 4/4 |
|  |  |  | ACCCCAAGG**CCT**TTG.**A**CCGAACGCCACGGAACTTTCTGCCATTGTTTGTT | +1 |  |
|  | **Line 10 (4424#10)** | chr15A | CTTGCTCCT**CCT**CCC.**T**TACTAGGCGGTGTGATGAAGTTTGTTAAAGC | +1 | 4/4 |
|  |  |  | ACCCCAAGG**CCT**TT**-**.CCGAACGCCACGGAACTTTCTGCCATTGTTTGTT | -1 |  |
|  |  | chr15D | CTTGCTCCT**CCT**CCC.**------------>** | -170 | 4/4 |
|  |  |  | **<------------**CGAACGCCACGGAACTTTCTGCCATTGTTTGTT |  |  |
|  |  | | | | |
|  | **WT (T89)** | chr15A | GCTCCTCCT**CCC**TAC.TAGGCGGTGTGATGAAGTTTGTTAAAGCATCCGG |  |  |
|  |  |  | AGGCCTTTG**CCG**AAC.GCCACGGAACTTTCTGCCATTGTTTGTTGATTGA |  |  |
|  |  | chr15D | GCTCCTCCT**CCC**TAC.TAGGCGGTGTGATGAAGTTTGTTAAAGCATCCGG |  |  |
|  |  |  | AGGCCTTTG**CCG**AAC.GCCACGGAACTTTCTGCCATTGTTTGTTGATTGA |  |  |
|  | **Line 35**  **(4440#35)** | chr15A | GCTCCTCCT**CCC**TAC.T***------------>*** | -171 | 3/3 |
|  |  |  | ***<------------***.GCCACGGAACTTTCTGCCATTGTTTGTTGATTGA |  |  |
|  |  | chr15D | GCTCCTCCT**CCC**TAC.**T**TAGGCGGTGTGATGAAGTTTGTTAAAGCATCCGG | +1 | 4/4 |
|  |  |  | AGGCCTTTG**CCG**AAC.**T**GCCACGGAACTTTCTGCCATTGTTTGTTGATTGA | +1 |  |
|  |  |  |  |  |  |
| **CRF3** | **WT (T89)** | chr12A | CTTGATCCT**CCT**CCC.TACTAGGCGGTGTGATGAAATTTGTTAAAGCATC |  |  |
|  |  |  | ACCCCAAGG**CCT**TTG.CCGAACGCCACGGAACTTTCTGCCATTGTTTGTT |  |  |
|  |  | chr12D | CTTGATCCT**CCT**CCC.TGCTAGGCGGTGTGATGAAATTTGTTAAAGCATC |  |  |
|  |  |  | ACCCCAAGG**CCT**TTG.CCGAACGCCACGGAACTTTCTGCCATTGTTTGTT |  |  |
|  | **Line 7**  **(4424#7)** | chr12A | CTTGATCCT**CCT**CCC.***------------>*** | -169 | 3/3 |
|  |  |  | ***<------------***.CCGAACGCCACGGAACTTTCTGCCATTGTTTGTT |  |  |
|  |  | chr12D | CTTGATCCT**CCT**CCC.***------------>*** | -150  +1 |  |
|  |  |  | ***<-------***TTACCCCAAGG**CCT**TTG**A**.CCGAACGCCACGGAACTTTCTGC |  |  |
|  | **Line 10 (4424#10)** | chr12A | CTTGATCCT**CCT**CCC.T***------------>*** | -168 | 4/4 |
|  |  |  | ***<------------***.CCGAACGCCACGGAACTTTCTGCCATTGTTTGTT |  |  |
|  |  | chr12D | CTTGATCCT**CCT**CCC.***------------>*** | -167 |  |
|  |  |  | ***<------------***TG.CCGAACGCCACGGAACTTTCTGCCATTGTTTGTT |  |  |
|  |  |  |  |  |  |
|  | **WT (T89)** | chr12A | GATCCTCCT**CCC**TAC.TAGGCGGTGTGATGAAATTTGTTAAAGCATCTGG |  |  |
|  |  |  | AGGCCTTTG**CCG**AAC.GCCACGGAACTTTCTGCCATTGGTGGTTGATTGG |  |  |
|  |  | chr12D | GATCCTCCT**CCC**TGC.TAGGCGGTGTGATGAAATTTGTTAAAGCATCTGG |  |  |
|  |  |  | AGGCCTTTG**CCG**AAC.GCCACGGAACTTTCTGCCATTGGTGGTTGATTGG |  |  |
|  | **Line 35**  **(4440#35)** | chr12A | GATCCTCCT**CCC**TAC.TAGGCGGTGTGATGAAATTTGTTAAAGCATCTGG | -138 | 4/4 |
|  |  |  | ***<---------------***TGCCATTGGTGGTTGATTGG |  |  |
|  |  | chr12D | GATCCTCCT**CCC**TGC.***------------>*** | -176 |  |
|  |  |  | ***<------------***CACGGAACTTTCTGCCATTGGTGGTTGATTGG |  |  |
